# Real-time gesture mapping system for a 15-DOF palm-scale bionic manipulator

**DOI:** 10.1371/journal.pone.0358347

**Published:** 2026-09-16

**Authors:** Qunting Chen, Xinhui Chen, Geng Chen

**Affiliations:** 1 College of Information Engineering, Jinhua University of Vocational Technology, Zhejiang, China; 2 College of New Energy Equipmnet, Zhejiang College of Security Technology, Zhejiang, China; National University of Sciences and Technology, PAKISTAN

## Abstract

In this paper, a low-cost, high-precision, high-degree-of-freedom intelligent manipulator was developed. Specifically, a 15-DOF palm-scale bionic manipulator integrated with a vision-based gesture mapping framework is presented. The system consists of a power management module, palm module, joint module, and gesture recognition module, and can be controlled via smart devices. Experimental results demonstrate gesture-driven control with an average end-to-end response time of approximately 2s, including perception, communication, and execution delays. In addition, the system is lightweight and compact, with dimensions comparable to those of a human palm. The work demonstrates the feasibility of integrating established gesture-recognition components with a compact robotic manipulator for embedded and portable human-robot interaction.

## 1. Introduction

Bionic manipulators have attracted extensive attention due to their wide applications in industrial production [1], medical surgery [2], prosthetics [3], and hazardous environment operations [4]. Despite significant progress, existing systems still face several critical challenges [4], including high manufacturing cost, limited degrees of freedom (DOF), insufficient motion precision, and lack of intelligent control capabilities. Among these issues, achieving a high-DOF design while maintaining system compactness and control efficiency remains particularly challenging [5].

Recent advances in robotic hands have emphasized high integration, dexterity, and perception capabilities [6–11]. High-DOF manipulators enable more human-like motion, while compact and lightweight designs improve portability and applicability in real-world scenarios [12–15]. Meanwhile, the integration of artificial intelligence into robotic systems has opened new opportunities for intuitive human–robot interaction, especially through vision-based gesture recognition [16–19]. Existing approaches, including image-based recognition, surface electromyography (sEMG) [20–22], and sensor gloves [23–25], have demonstrated promising results. However, these methods often suffer from high hardware cost, computational complexity, or limited adaptability to embedded platforms.

Although deep learning-based gesture recognition methods, such as convolutional neural networks (CNN [26]) and 3D CNN [27], provide high accuracy, they typically require substantial computational resources, making them less suitable for lightweight and portable systems. On the other hand, traditional methods lack robustness and scalability in dynamic environments. Therefore, there is a strong need for a low-cost, efficient, and real-time gesture-driven control framework that can be seamlessly integrated with high-DOF manipulators.

To address these challenges, this paper presents a lightweight intelligent manipulator system that integrates a 15-DOF palm-scale bionic hand with a real-time gesture recognition and control framework. The 21-keypoint detection and ST-GCN components are adopted from established vision-based gesture-recognition approaches. Accordingly, the primary contribution of this work is system-level integration: combining vision-based perception, gesture classification, wireless communication, embedded control, and multi-joint actuation in a compact, low-cost prototype.

The main contributions of this work are summarized as follows:

1 We develop and experimentally validate a compact 15-DOF palm-scale bionic manipulator with an integrated embedded control architecture, emphasizing compactness and portability.
2 We integrate an established vision-based gesture-recognition pipeline using 21-keypoint detection and a Spatio-Temporal Graph Convolutional Network (ST-GCN). The purpose is to provide an effective perception component for the manipulator.
3 We implement an end-to-end perception–communication–actuation pipeline and experimentally characterize its response time, with an average reported value of approximately 2 s.
4 We demonstrate the integration of an ESP32S3-based embedded controller with a commodity smartphone, providing a low-cost prototype for gesture-driven human–robot interaction. Claims regarding specific field deployment scenarios are treated as potential applications.

The remainder of this paper is organized as follows. Section 2 reviews related work. Section 3 describes the system design and methodology. Section 4 presents experimental results and analysis. Finally, Section 5 concludes the paper.

## 2. Related Works

### 2.1. Virtual servo drive circuit

The fundamental reason why a robotic hand can mimic various human hand postures lies in its incorporation of multiple servos, and of course, the corresponding servo drive circuits. To precisely control the rotation angle of each finger, the accuracy in controlling servo positions becomes particularly crucial. Petrescu [28] described a hybrid control method for a two-phase stepper motor, primarily using PID combined with neural networks to achieve intelligent and precise control of the stepper motor. However, for a robotic hand requiring multi-joint coordination, such a combined approach is not conducive to rapid movement. Enaje [29] employed the A4988 as the drive chip for a micro motor, achieving serial port control of the motor drive module to drive a micro stepper motor with precise control over speed and step angle. Nevertheless, the step resolution of this drive chip remains relatively limited. Therefore, we have selected the A4950 as the drive chip for each joint to achieve finer and smoother hand movements.

### 2.2. Gesture recognition

Gesture recognition algorithms are at the core of the fields of computer vision and pattern recognition. They typically serve as the “brain” of the entire gesture recognition system, processing and interpreting data collected by sensors. These algorithms can be broadly categorized into two main groups based on whether they follow traditional or modern approaches, namely traditional machine learning methods and deep learning methods.

Traditional machine learning methods generally follow a “feature extraction + classifier” pipeline, heavily relying on manually designed features. Dynamic Time Warping (DTW) [30,31] is a popular algorithm within this category. It compares the trajectory or contour of a gesture to be recognized with predefined standard templates to find the best match. However, when the number of templates increases, the computational cost for matching rises significantly. The Hidden Markov Model (HMM) [32,33] is another classic algorithm in this category. It treats a gesture sequence as a process where states change over time, modeling and recognizing gestures through probabilistic models. HMM is particularly well-suited for capturing the temporal characteristics of dynamic gestures but requires a large amount of annotated data for model training.

Deep learning methods are currently the mainstream approach, capable of learning an end-to-end mapping from data to results automatically. The feature extraction process in deep learning is implicit and data-driven. Among these methods, the 3D Convolutional Neural Network (3D CNN) [34,35] algorithm employs 3D convolution kernels to operate simultaneously in spatial (width, height) and temporal dimensions, learning spatiotemporal features directly from video clips. This enables better capture of motion information in gestures. However, it places extremely high demands on hardware and is not easy to deploy on mobile or embedded devices. In contrast, the 2D Convolutional Neural Network (2D CNN) [36,37] algorithm takes each frame of a gesture image or a keypoint heatmap as input and uses CNN to extract spatial features. For dynamic gestures, it can process stacked multiple frames or aggregate results from per-frame predictions. This algorithm can automatically learn visual patterns from low-level to high-level features. Moreover, the technology is mature, with numerous pre-trained models (e.g., ResNet, MobileNet) available for transfer learning.

Recent studies have demonstrated the effectiveness of deep learning-based perception systems in real-world applications. For example, FE-SpikeFormer introduces a camera-based facial expression recognition framework for healthcare monitorin [38], while dual-pathway emotion recognition networks further validate the robustness of multi-task learning strategies in dynamic environments [39]. These works highlight the practicality and scalability of deep learning-based recognition systems.

To enable accurate gesture recognition while avoiding substantial hardware costs, we adopt a 21-keypoint gesture-recognition approach [40]—a combined framework utilizing a CNN-based keypoint detection model and a Spatio-Temporal Graph Convolutional Network (ST-GCN). In this work, these components are used as established building blocks within the proposed manipulator system.

## 3. Methodology

### 3.1. System architecture

**Fig 1:**
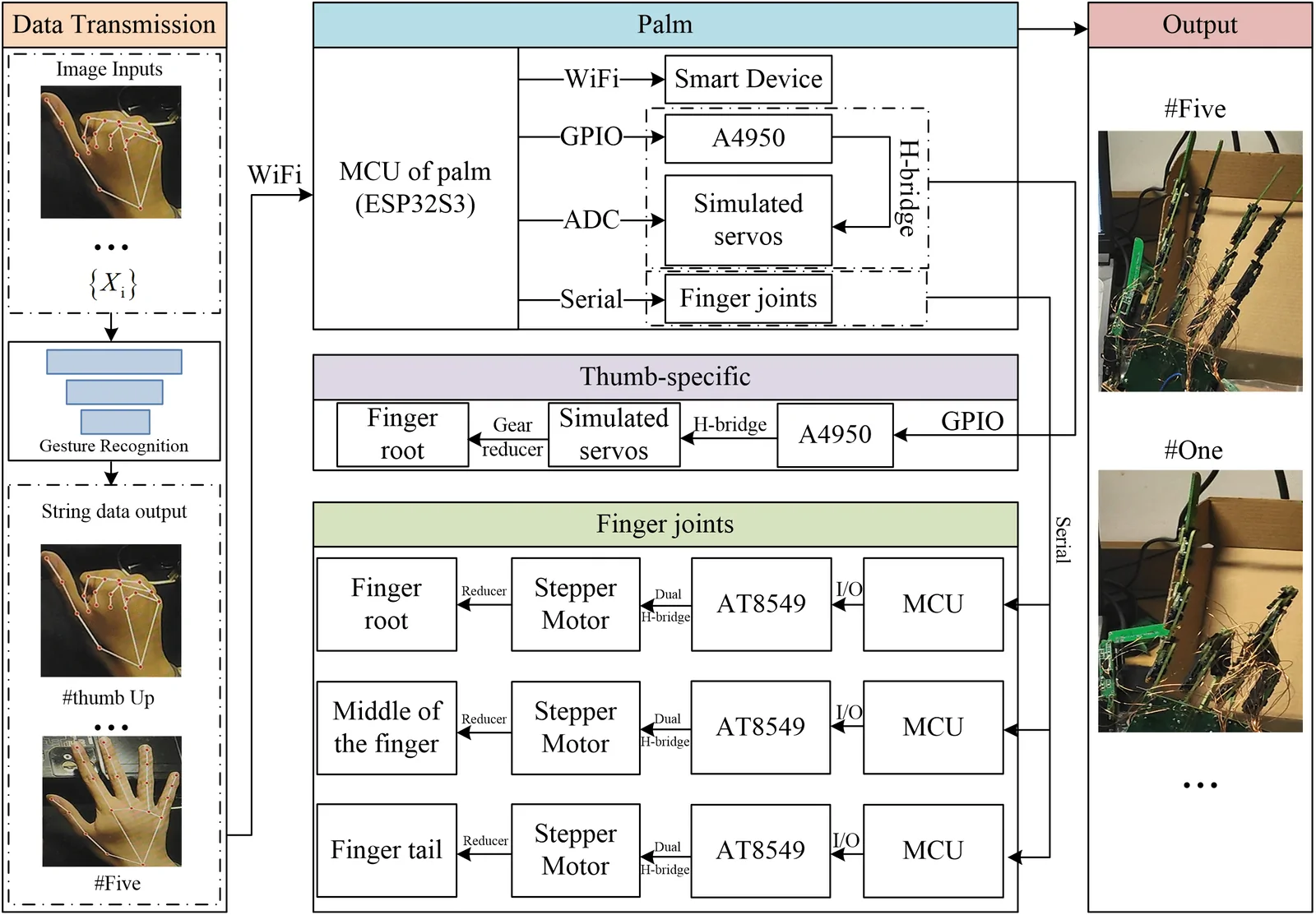
System architecture of the proposed gesture-driven manipulator. The system includes a smart device for gesture recognition, an ESP32S3-based control module, and a 15-DOF bionic manipulator, enabling wireless transmission and execution of gesture commands.

The overall architecture diagram is shown in Fig 1A, and the system mainly consists of data transmission, power management module and palm module. The fingers module in the system is used to receive UART commands to control fourteen screw stepper motors to carry out the movement of the fingers. The system uses a charger that supports PD3.0 to output a power supply of 36W to power the palm module and fingers module in the system. The palm module uses ESP32S3 as a smart devices terminal to receive, process, and forward the data streams to the fingers module. The smart device builds a Linux environment through AidLux to run Python scripts to obtain camera images and implement gesture recognition through the Mediapipes framework. The overall mass does not exceed 0.3 kg and the volume is 12 cm*20 cm*4 cm, so it is a portable device and highly suitable for deployment in diverse application scenarios.

As shown in Fig 1, each finger has three sets of curved joints except the thumb. The thumb consists of a rotating joint as the root and two curved joints as the middle and tail of the fingers.

The rotary joint motion of the fingers is achieved by controlling the angle of the simulated servo through the ADC and GPIO in the ESP32S3 of palm module. The bending joints of the fingers receive the data sent by the palm through the UART of PY32F002A, which connects with the palm. The GPIO pins then control the stepper motor to drive the planetary reduction screw to achieve telescopic movement, and enable finger bending motion through the second-class bar group.

The palm structure is shown in Fig 1, ESP32S3 is used to identify and forward data streams from the mobile phone and distribute them to the finger joints. On the other hand, 5-wire analog servos are also controlled by GPIO and ADC of ESP32S3.

### 3.2. Software design of finger joints

After the system is powered on, the MCU of the palm assigns a unique ID to each joint. Then, the stepper motor is reset, that is, the palm extension action, to ensure that each joint ID is correctly assigned and can be driven normally. Once the MCU of joints obtains the UART commands, it identifies the ID of the data head to determine whether it corresponds to valid control commands and data of the joint, and then parse the commands and data to control the stepper motor to the corresponding stroke, and continues operation until system shutdown.

**Fig 2:**
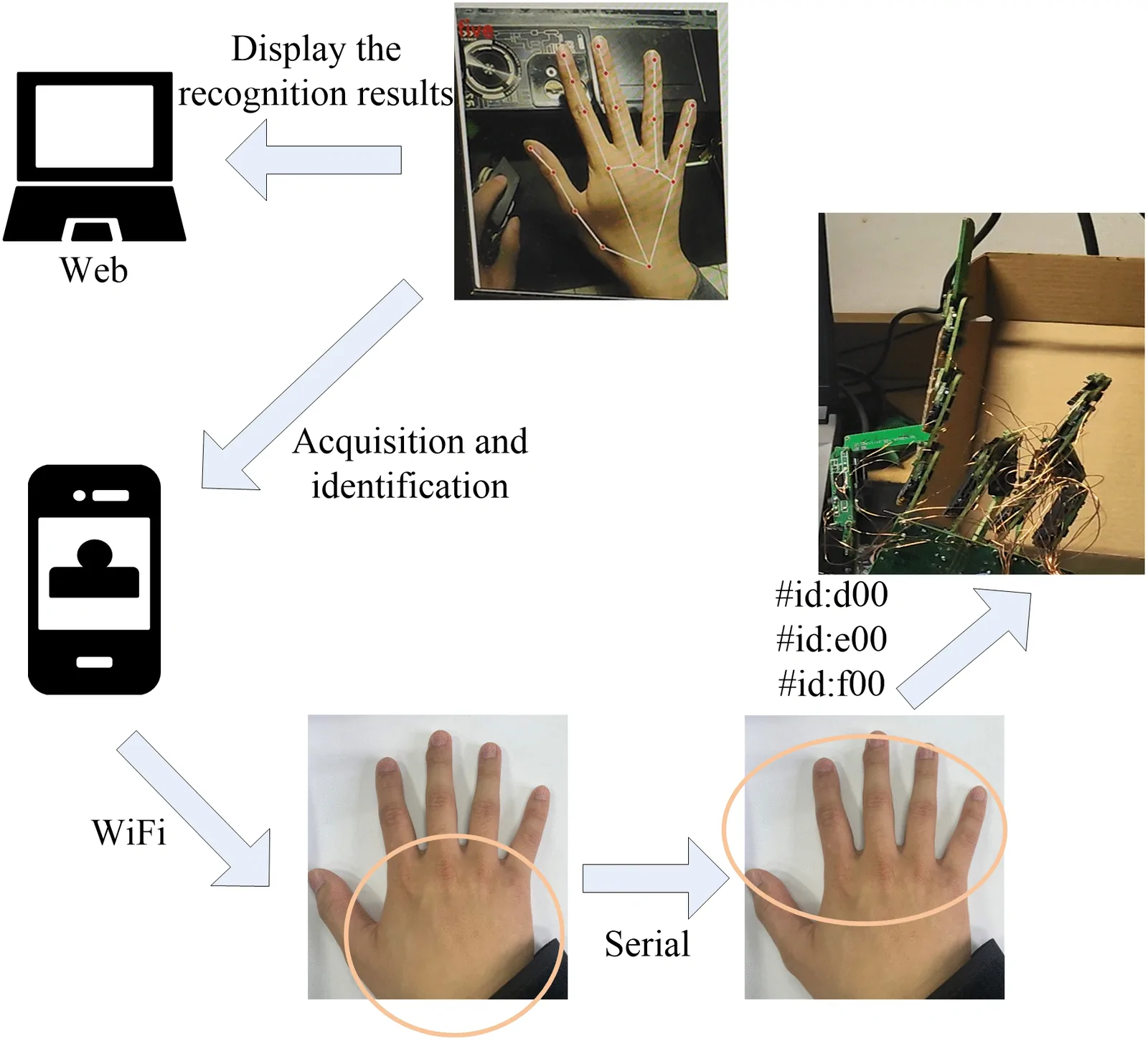
Software structure and communication protocol. The software framework defines data processing and UART-based communication between the palm controller and finger joints using a lightweight Modbus protocol.

As shown in Fig 2, the software structure defines the data processing and communication flow of the system. In order to ensure the reliability of the data, a simple Modbus [25] communication protocol is proposed, with the frame header data of ‘#,’ followed by the ID number of each joint and the percentage value of the stroke data, and finally ‘;’ is used as the end of the data frame. If you want the index finger of manipulator to stand up, the palm master control chip only needs to send “#id:d00; #id:e00; #id:f00;” to the index finger master control chip through the serial port.

### 3.3. Palm software design

After the system is powered on, the main control chip ESP32S3 reset the thumb rotation joint, and then connect to the network and wait for the network data. At present, the manipulator can complete the function in the description column as shown in Table 1, and when receiving the data in the table, the main control chip ESP32S3 will send the corresponding data to each finger or control the rotating joint of the thumb.

**Table 1:** Detection, identification and forwarding of palm receiving data.

Data received	Forward to the thumb	Forwarded to the index finger	Forward to the middle finger	Forward to the ring finger	Forward to the little finger	description
#five;	#id:a00; #id:b00; #id:c00;	#id:d00; #id:e00; #id:f00;	#id:g00; #id:h00; #id:i00;	#id:j00; #id:k00; #id:l00;	#id:m00; #id:n00; #id:o00;	Extend the palm
#fist;	#id:a75; #id:b75; #id:c75;	#id:d75; #id:e75; #id:f75;	#id:g75; #id:h00; #id:i75;	#id:j75; #id:k75; #id:l75;	#id:m75; #id:n75; #id:o75;	Fist
#thumb Up;	#id:a75; #id:b75; #id:c75;	#id:d00; #id:e00; #id:f00;	#id:g00; #id:h00; #id:i00;	#id:j00; #id:k00; #id:l00;	#id:m00; #id:n00; #id:o00;	Thumbs up
#one;	#id:a00; #id:b00; #id:c00;	#id:d75; #id:e75; #id:f75;	#id:g00; #id:h00; #id:i00;	#id:j00; #id:k00; #id:l00;	#id:m00; #id:n00; #id:o00;	Raise the index finger

## 4. Experiments

### 4.1. Experimental Setup

The experimental platform consists of a 15-DOF palm-scale bionic manipulator, an ESP32S3-based control module, and a smartphone (OPPO Reno4 5G) serving as the vision and interaction terminal. The smartphone captures hand gesture images and performs real-time gesture recognition using a 21-keypoint detection framework combined with ST-GCN. A total of 20 participants participated in the experiment. Four gesture classes, namely Extend the palm, Fist, Thumbs up, and Raise the index finger, were evaluated. Each participant performed each gesture 50 times, resulting in 1000 samples in total. And we divided the 1532 dynamic gestures included in NVGesture into 1050 training samples and 482 test samples.

The recognized gesture data are transmitted to the manipulator via Wi-Fi. The system processes gesture recognition, communication, and actuation in an end-to-end pipeline.

To evaluate system performance, we consider recognition accuracy, end-to-end response time, joint-level motion performance, and hardware/deployment characteristics.

### 4.2. Motor Performance Evaluation

To verify the basic actuation capability of the manipulator, we conducted joint-level motion tests. Control commands were sent directly to the palm controller via a desktop computer.

The results are summarized in Table 2. The manipulator demonstrates stable motion across all 15 degrees of freedom, with joint rotation angles ranging from 50° to 91°. The execution time for individual joint movement is typically between 0.3 s and 1 s.

**Table 2:** The angle at which each joint can be moved and the time performance.

Finger Joint	Thumb	Index finger	Middle finger	Ring finger	Little finger
Root joints	70°, 0.3s	86°, 0.4s	91°, 0.4s	88°, 0.4s	80°, 1s
Middle joints	78°, 0.4s	70°, 1s	80°, 0.4s	86°, 0.4s	86°, 0.4s
Fingertip joints	85°, 0.4s	50°, 1s	85°, 0.4s	70°, 0.3s	87°, 0.4s

These results demonstrate stable joint-level motion capability and provide evidence for the feasibility of the proposed mechanical and control design. However, grip force, payload capacity, and manipulation repeatability were not measured in the present experiments and therefore are not claimed here.

### 4.3. System-Level Functional Evaluation

To evaluate the overall system performance, real-time gesture control experiments were conducted. The smartphone captures gesture images and transmits recognized keypoint data to the manipulator.

**Fig 3:**
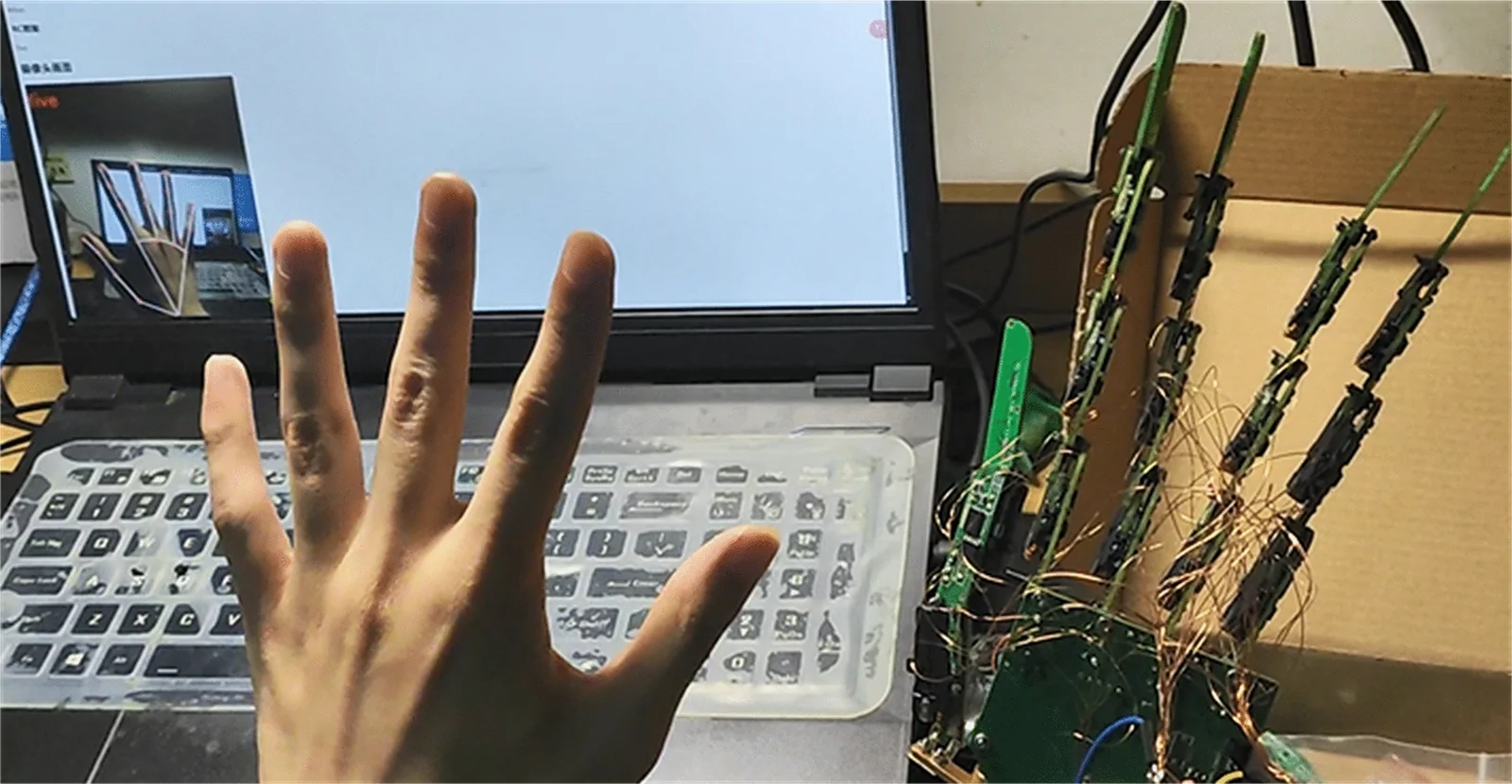
Gesture recognition and reproduction results on the manipulator. The system captures human gestures and reproduces them on the manipulator, demonstrating real-time performance and stable gesture mapping.

As shown in Fig 3, the system successfully reproduces human hand gestures on the manipulator. The reported average end-to-end response time is approximately 2 s, including 0.3 s for gesture recognition and data preprocessing, 0.7 s for wireless communication, and 1.0 s for mechanical execution. These values should be interpreted as average measurements from the reported evaluation; variability across repeated trials should be added as mean ± standard deviation when the underlying trial-level measurements are available.

### 4.4. Comparative Analysis

To contextualize the proposed system, Table 3 compares it with representative gesture-recognition and control approaches reported in the literature. The values for DTW-, HMM-, CNN-, and 3D CNN-based approaches were not reproduced on the same hardware and dataset in the present study.

**Table 3:** Contextual comparison with representative gesture-controlled manipulator systems.

Method	DOF	Recognition Method	Accuracy (%)	Response Time (s)	Hardware Cost	Experimental basis
DTW-based [41]	10	Template matching	82.5	3.5	Low	Reported in [41]
HMM-based [42]	12	Probabilistic model	85.3	3.2	Medium	Reported in [42]
CNN-based^[26]^	15	2D CNN	91.2	2.8	High	Reported in [26]
3D CNN-base [27]	15	3D CNN	93.5	4.0	Very High	Reported in [27]
**This work**	**15**	**Keypoint + ST-GCN**	**92.6**	**2.0**	**Low**	**This study**

The literature-reported methods provide useful context for the design trade-offs considered in this work, but differences in datasets, hardware platforms, preprocessing procedures, and evaluation protocols limit direct quantitative comparison. The proposed system reports 92.6% accuracy and a 2.0 s end-to-end response time under the experimental conditions described in this manuscript. These results demonstrate the feasibility of the integrated prototype.

### 4.5. Ablation Study

To investigate component contributions, we report the available component comparisons in Table 4. The comparison labeled “Without Keypoint (CNN only)” changes both the input representation and the recognition architecture and therefore is not a single-factor ablation. It is retained as an alternative architecture baseline rather than being interpreted as an isolated measurement of the keypoint contribution. A clean one-factor ablation should be added in a subsequent experimental revision by changing only one component at a time while keeping the dataset, input representation, training protocol, and evaluation hardware fixed.

**Table 4:** Ablation study of the proposed method.

Configuration	Accuracy (%)	Response Time (s)	Computation Cost
Full Model (Keypoint + ST-GCN)	92.6	2.0	Low
Raw-image CNN baseline (input + architecture changed)	88.1	1.8	Low
Without Keypoint (CNN only)	90.3	2.8	High
Keypoint + Simple Classifier	89.5	2.1	Low

As shown in Table 4, the full model achieves the highest reported accuracy among the listed configurations. Removing ST-GCN changes the reported accuracy from 92.6% to 88.1%, providing evidence that the temporal modeling component is useful under the tested configuration. However, because the other reduced configurations may alter more than one factor, the table should be interpreted as component/architecture comparison rather than a strictly isolated ablation.

The “Without Keypoint (CNN only)” configuration changes both the input representation and the model architecture; therefore, its increase in response time to 2.8 s and its accuracy of 90.3% cannot be attributed solely to removing keypoint representation. Similarly, the “Keypoint + Simple Classifier” configuration provides an architecture comparison. Overall, these results support the feasibility of the complete pipeline, while a controlled one-factor ablation is required to quantify the independent contribution of each component.

## 5. Limitations and Future Work

Although the proposed system demonstrates the feasibility of vision-based gesture control for a 15-DOF palm-scale bionic manipulator, several limitations remain.

**First, the current evaluation uses a single smartphone model (OPPO Reno4 5G).** The effects of different cameras, processing capabilities, and device-specific latency have not been systematically evaluated. In addition, the robustness of gesture recognition under different illumination, backgrounds, camera viewpoints, and hand-occlusion conditions remains to be investigated. Future work will evaluate the system across multiple devices and more challenging environmental conditions.

**Second, the current gesture vocabulary contains four predefined gestures: Extend the palm, Fist, Thumbs up, and Raise the index finger.** Although these gestures are sufficient to demonstrate the proposed control framework, they do not constitute a general-purpose gesture-recognition system. Future studies will expand the gesture vocabulary and evaluate cross-user generalization, including users with different hand sizes and anthropometric characteristics.

**Third, the mechanical performance of the manipulator has not been comprehensively characterized.** Grip force, payload capacity, positioning accuracy, repeatability, and manipulation success rate were not systematically measured in the present study. Future work will establish standardized mechanical tests to quantitatively evaluate these properties.

**Fourth, the current prototype is primarily a laboratory proof-of-concept system.** The exposed wiring and temporary mounting structure facilitate prototyping and debugging but are not suitable for long-term or field deployment. Future iterations will improve mechanical integration, cable management, structural protection, and operational safety.

**Finally, the potential applications in law enforcement, disaster response, and hazardous-object handling should be regarded as prospective applications rather than experimentally validated capabilities.** The present study does not include hazardous-object manipulation or comprehensive field safety testing. Future work will therefore focus on statistically controlled experiments, cross-user and cross-device evaluation, mechanical characterization, and safety validation before considering practical deployment.

Overall, this work should be regarded as a **low-cost prototype demonstrating the feasibility of integrating vision-based gesture recognition with a palm-scale 15-DOF bionic manipulator.** Future research will focus on improving recognition robustness, expanding the gesture vocabulary, reducing system latency, and enhancing the mechanical reliability and safety of the platform.

## 6. Conclusions

This paper presents a lightweight intelligent bionic manipulator system integrating a 15-DOF palm-scale robotic hand with an vision-based gesture-driven control framework. The main contribution is the system-level integration of established gesture-recognition components with embedded control and multi-joint actuation in a compact prototype.

Experimental results demonstrate an average gesture recognition accuracy of 92.6% and an end-to-end response time of approximately 2.0 s, including 0.3 s for perception, 0.7 s for communication, and 1.0 s for actuation. At the hardware level, all 15 degrees of freedom exhibit stable joint motion, with response times ranging from 0.3 s to 1.0 s and rotation angles up to 91°. These results characterize the prototype under the tested condition.

Compared with representative methods, the proposed approach provides a favorable trade-off between accuracy, response latency, and hardware cost. The 28.6% response-time reduction is obtained relative to the cited CNN-based result (2.8 s) and is calculated as (2.8 − 2.0) / 2.8 × 100% = 28.6%.

The system demonstrates successful real-time gesture reproduction in the tested setup, indicating the feasibility of vision-based human–robot interaction. The use of an ESP32S3 and a commodity smartphone supports the low-cost prototype objective.

The gesture-driven control architecture may have potential relevance to remote manipulation and other human–robot interaction scenarios in which intuitive teleoperation is desirable [43]. However, applications such as explosive ordnance disposal, hazardous-object handling, disaster response, and policing [44,45] have not been experimentally validated in this study. In particular, no hazardous-object handling, payload, grip-force, safety, or field-deployment tests were conducted. These scenarios should therefore be regarded as potential future applications.

Future work will focus on improving recognition robustness under complex environment, reducing system latency, expanding the gesture vocabulary, performing statistically controlled comparisons and one-factor ablations, evaluating cross-user and cross-device generalization, and characterizing grip force, payload capacity, repeatability, and deployment safety.

## Supporting information

S1 File Raw_images. This PDF file contains all the figures in the article.
(PDF)

S2 File Android-aidlux. This folder contains the webapp introduced in this document. It can be run using Python in the conda environment.
(ZIP)

S3 File Arduino-ESP32S3. This folder contains the programs that are running on the ESP32S3 in this system. Readers can open and edit these programs in the Arduino IDE software.
(ZIP)

S4 File JLCeda-PCB. This folder contains the circuit schematic diagrams and PCB layouts used in the creation of this system. We can open them using Altium Designer.
(ZIP)

S5 File keil-PY32F003A. This folder contains the programs running on the main control chip of this system. Readers can use the Keil IDE software to open or edit them.
(ZIP)
