# Supplementary material for: Real-time gesture mapping system for a 15-DOF palm-scale bionic manipulator
Source: PLoS One. 2026 Sep 16;21(9):e0358347. doi: 10.1371/journal.pone.0358347 (PMC13580999; doi:10.1371/journal.pone.0358347)
Supplement: Supplementary file 1 [file pone.0358347.s001.pdf]

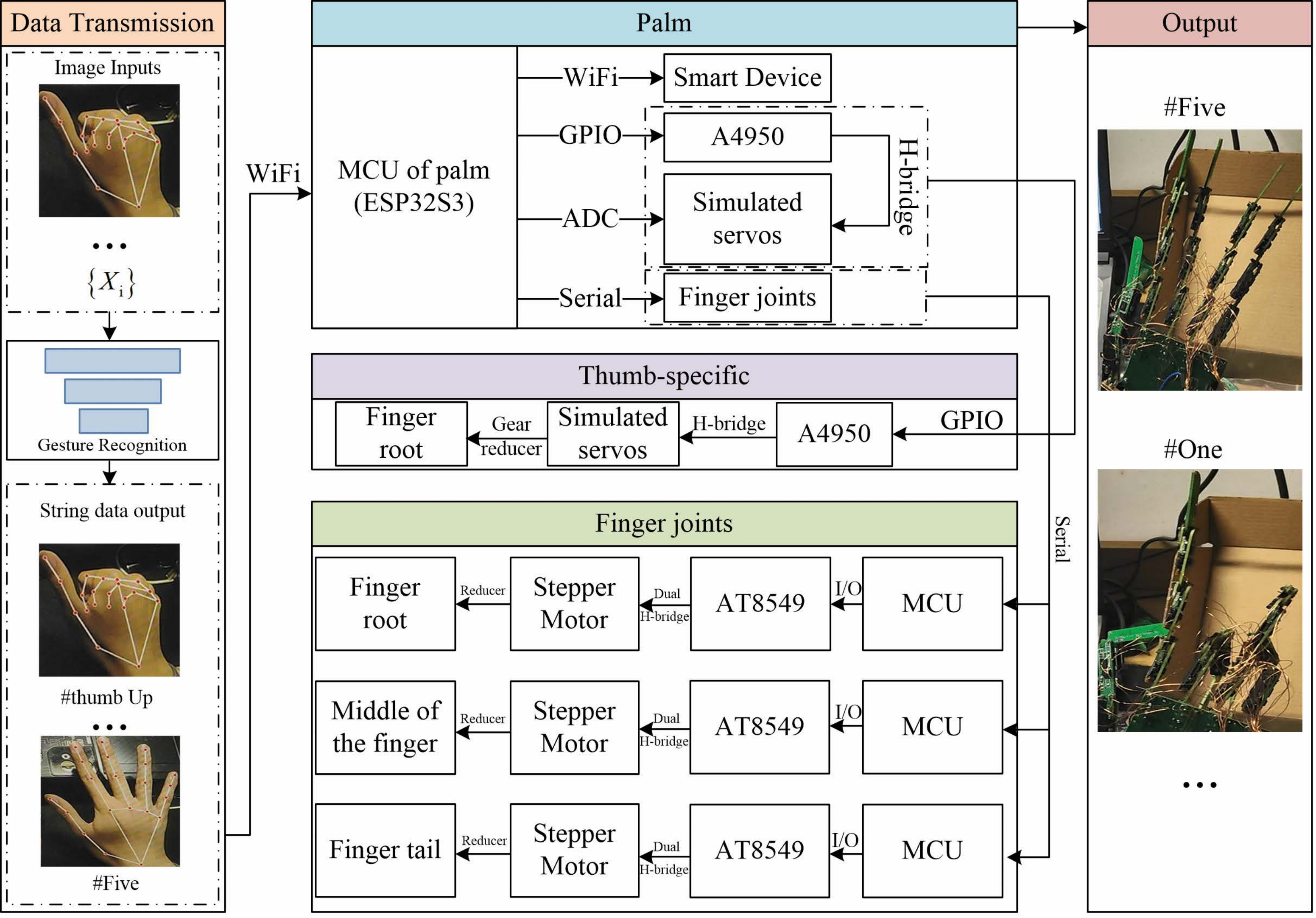

Fig 1. System architecture of the proposed gesture-driven manipulator

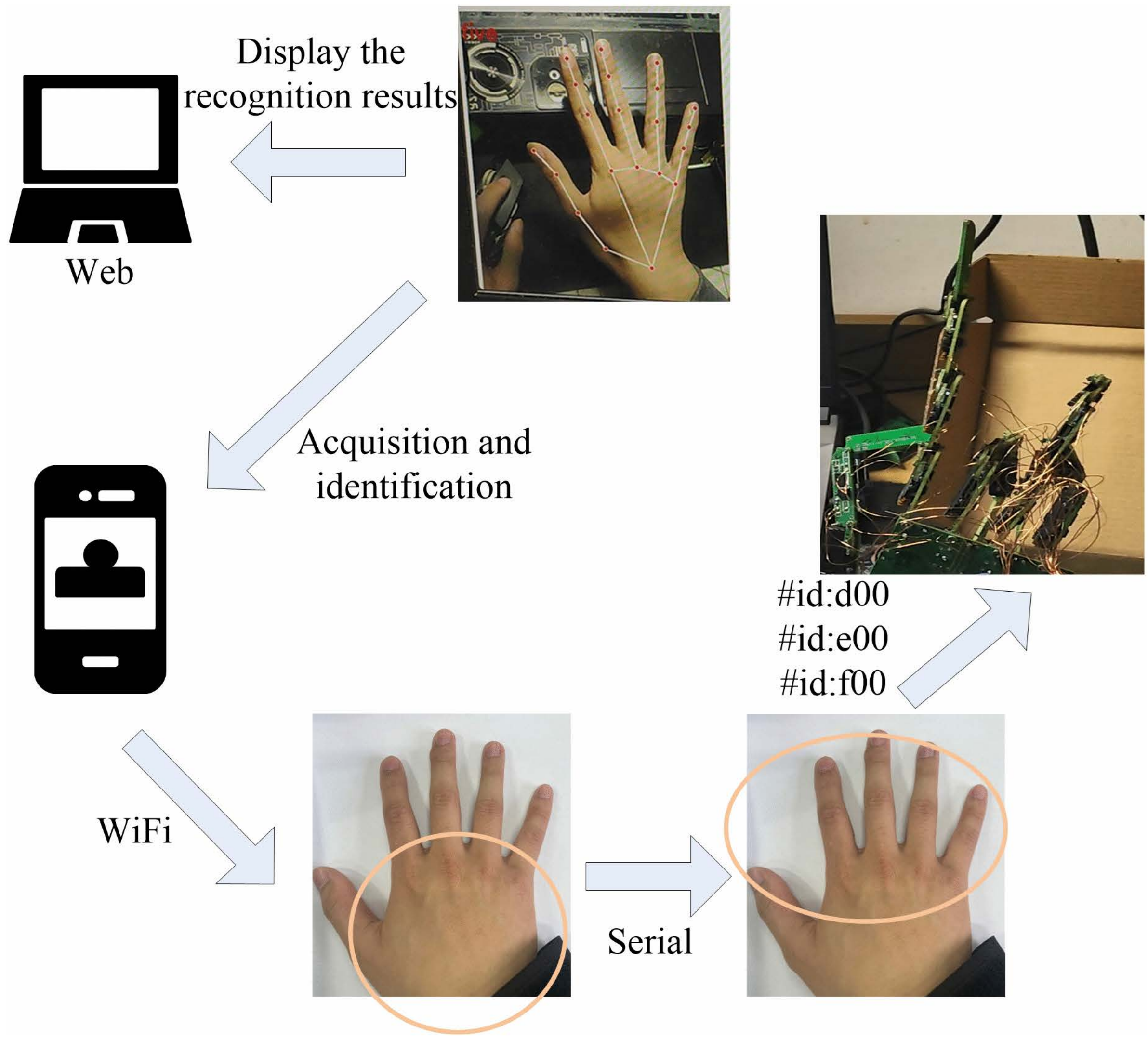

Fig 2. Software structure

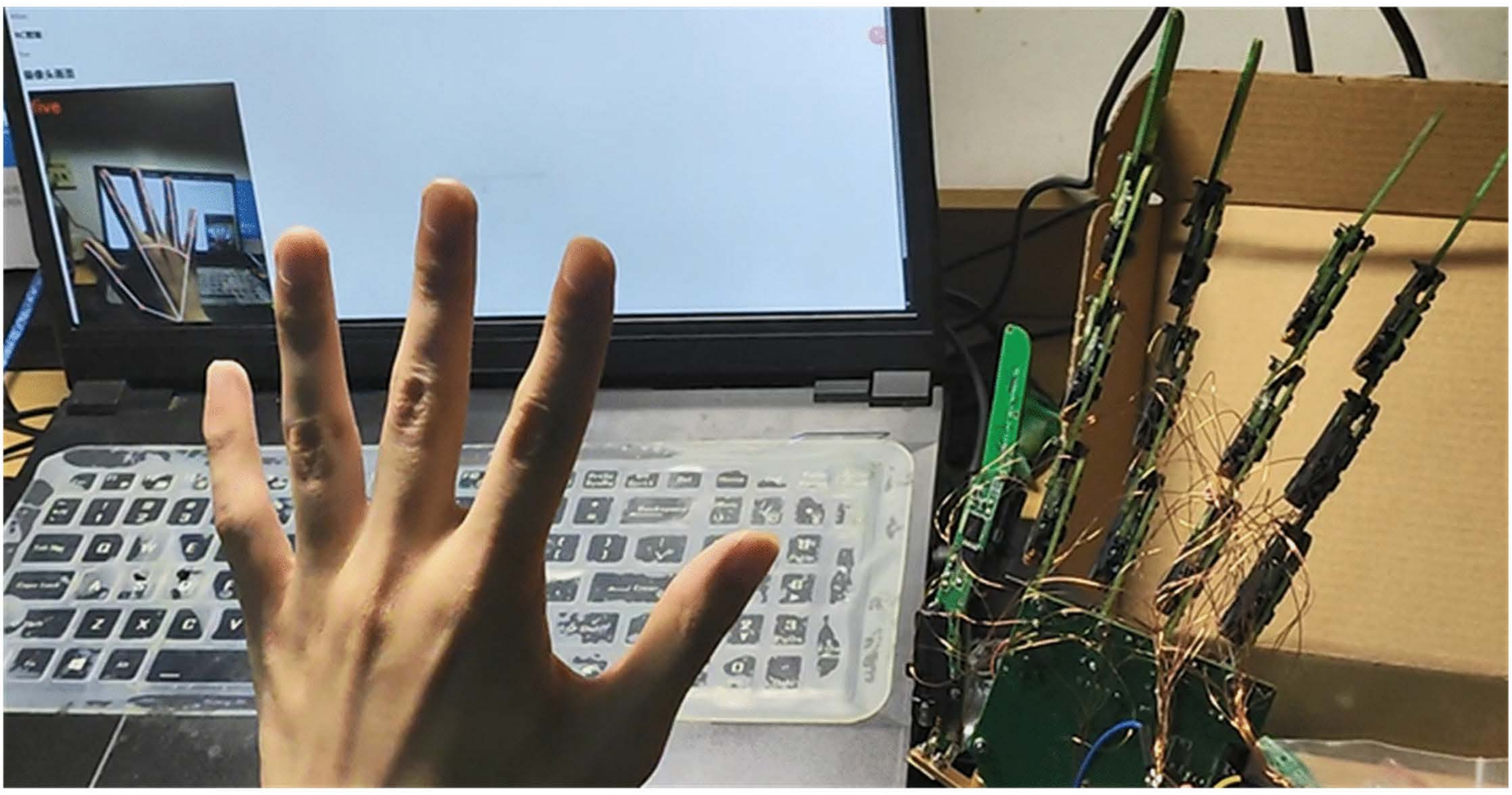

Fig 3. Gesture recognition and reproduction results on the manipulator
