# Supplementary material for: Real-time gesture mapping system for a 15-DOF palm-scale bionic manipulator
Source: PLoS One. 2026 Sep 16;21(9):e0358347. doi: 10.1371/journal.pone.0358347 (PMC13580999; doi:10.1371/journal.pone.0358347)
Supplement: Supplementary file 2 [file pone.0358347.s007.docx]

Dear Reviewer,

We appreciate reviewer’s comments and valuable queries, which have helped us improve the clarity and quality of our manuscript. Given below are detailed point-by-point responses to the questions and suggestions. The corresponding modifications are incorporated in the revised manuscript (highlighted in yellow). We believe our manuscript has been improved significantly and now deserves publication in PLOS ONE.

Yours sincerely

Qunting Chen on behalf of all co-authors

Reviewers’ comments:

Reviewer #1: This paper presents a gesture mapping system for a 15-DOF palm-scale bionic manipulator. The topic is interesting, and the paper’s topic is also highly aligned with the academic programs of this institution. While some key issues should be further clarified below:

**Response)** We appreciate the reviewer’s comments “*The topic is interesting*”. We respond to the reviewer’s comments described below, which will hopefully alleviate the reviewer’s concerns.

(1) The paper’s innovations should be more clearly distilled, ideally by grounding them in the fundamental bottlenecks of the field and formulating targeted solutions accordingly.

**Response)** We appreciate the reviewer for this important question. In the revised manuscript, we clearly identified the key challenges in high-DOF manipulator control in page 2-3.

These revisions significantly improve the clarity and emphasis of the paper’s novelty.

*“Although deep learning-based gesture recognition methods, such as convolutional neural networks (CNN[26]) and 3D CNN[27], provide high accuracy, they typically require substantial computational resources, making them less suitable for lightweight and portable systems. On the other hand, traditional methods lack robustness and scalability in dynamic environments. Therefore, there is a strong need for a low-cost, efficient, and real-time gesture-driven control framework that can be seamlessly integrated with high-DOF manipulators.*

*To address these challenges, this paper proposes a lightweight, AI-enhanced intelligent manipulator system that integrates a 15-DOF palm-scale bionic hand with a real-time gesture recognition and control framework. Compared with existing studies, the proposed system emphasizes the balance among system complexity, real-time performance, and hardware cost, making it particularly suitable for embedded and portable applications.*

*The main contributions of this work are summarized as follows:*

*1)	We propose a compact 15-DOF palm-scale bionic manipulator is developed with a highly integrated structure, enabling both dexterity and portability.*

*2)	We introduce a lightweight vision-based gesture recognition framework is designed by combining 21-keypoint detection with a spatio-temporal graph convolutional network, which enables efficient and accurate recognition on resource-constrained devices.*

*3)	We conduct a complete end-to-end system integrating perception, communication, and actuation is implemented, achieving stable real-time control with an average response time of approximately 2 seconds.*

*4)	The system is built using embedded hardware (ESP32S3) and commodity smart devices, providing a practical and scalable solution for real-world applications such as remote manipulation and human–robot interaction.”*

(2) Although the techniques in this paper are practical and relatively mature, the authors are encouraged to cite more relevant literature (FE-SpikeFormer: A Camera-based Facial Expression Recognition method for Hospital Health Monitoring； A Dual-Pathway Driver Emotion Classification Network Using Multi-Task Learning Strategy: A Joint Verification) to demonstrate their effectiveness and adoption in real-world applications.

**Response)** We feel sorry that the number of relevant references does not meet the requirements. We list some important relevant literature as below.

*“[38]	Dong Z, Zhu L, Zhou S, et al. FE-SpikeFormer: A Camera-Based Facial Expression Recognition Method for Hospital Health Monitoring[J]. IEEE Journal of Biomedical and Health Informatics, 2025:1-11.*

*[39]	Z. Dong, C. Hu, L. Zhu, et al. A Dual-Pathway Driver Emotion Classification Network Using Multitask Learning Strategy: A Joint Verification[J]. IEEE Internet of Things Journal, 2025, 12(10):14897-14908.”*

In particular, we have incorporated references to FE-SpikeFormer, a camera-based recognition system, and dual-pathway emotion classification networks. These works are discussed in Section 2.2 to highlight the practical effectiveness of deep learning-based perception systems in real-world applications.

“*Recent studies have demonstrated the effectiveness of deep learning-based perception systems in real-world applications. For example, FE-SpikeFormer introduces a camera-based facial expression recognition framework for healthcare monitorin[38], while dual-pathway emotion recognition networks further validate the robustness of multi-task learning strategies in dynamic environments[39]. These works highlight the practicality and scalability of deep learning-based recognition systems.*”

(3) The major weakness of the paper lies in the insufficient comparative experiments. The authors are encouraged to add more comprehensive comparisons; doing so would better highlight the strengths of the proposed method and significantly improve the completeness of the work.

**Response)** We sincerely appreciate this important comment. In response, we have strengthened the experimental validation in several ways. First, we added a new comparative experiment in Table 3, where our method is evaluated against DTW, HMM, CNN, and 3D CNN baselines. Second, we introduced a new discussion subsection in Section 4.4 to analyze the performance differences in greater detail. Third, we included quantitative metrics such as accuracy, response time, and hardware cost to provide a more comprehensive evaluation.

**Table 3. Comparison with existing gesture-controlled manipulator systems**

| **Method** | **DOF** | **Recognition Method** | **Accuracy (%)** | **Response Time (s)** | **Hardware Cost** |
| --- | --- | --- | --- | --- | --- |
| DTW-based^[41]^ | 10 | Template matching | 82.5 | 3.5 | Low |
| HMM-based^[42]^ | 12 | Probabilistic model | 85.3 | 3.2 | Medium |
| CNN-base^[26]^ | 15 | 2D CNN | 91.2 | 2.8 | High |
| 3D CNN-based^[27]^ | 15 | 3D CNN | 93.5 | 4.0 | Very High |
| **Proposed Method (Ours)** | **15** | **Keypoint + ST-GCN** | **92.6** | **2.0** | **Low** |

*“ [26]Trelinski J, Kwolek B. CNN-based and DTW features for human activity recognition on depth maps[J]. Neural Comput & Applic, 2021, 33:14551–14563*

*[27]Funke I, Bodenstedt S, Oehme F, et al. Using 3D Convolutional Neural Networks to Learn Spatiotemporal Features for Automatic Surgical Gesture Recognition in Video[J]. MICCAI 2019, 2019:467-475.*

*[41]	Schneider P, Memmesheimer R, Kramer, et al. Gesture Recognition in RGB Videos UsingHuman Body Keypoints and Dynamic Time Warping[J]. RoboCup 2019: Robot World Cup XXIII, 2019: 281–293*

*[42]	Chen F S, Fu C M, Huang C L. Hand gesture recognition using a real-time tracking method and hidden Markov models[J]. Image and Vision Computing, 2003, 21(8):745-758. ”*

In addition, we further enhanced the experimental section by adding an ablation study in Table 4 to assess the contributions of the key components, including ST-GCN and the keypoint representation. These revisions substantially improve the completeness and rigor of the experimental validation.

**Table 4. Ablation study of the proposed method**

| **Configuration** | **Accuracy (%)** | **Response Time (s)** | **Computation Cost** |
| --- | --- | --- | --- |
| Full Model (Keypoint + ST-GCN) | 92.6 | 2.0 | Low |
| Without ST-GCN | 88.1 | 1.8 | Low |
| Without Keypoint (CNN only) | 90.3 | 2.8 | High |
| Keypoint + Simple Classifier | 89.5 | 2.1 | Low |

(4) It is recommended that the paper provide some application visions that connect the proposed work with practical policing scenarios, in order to enhance its real-world relevance.

**Response)** Thank you very much for this insightful and valuable suggestion. We fully agree that connecting the proposed work with practical policing scenarios would further enhance its real-world relevance. Although we were unable to carry out additional application-specific experiments due to the current scope and limitations of our study, we have made our best effort to enrich the manuscript by adding a more in-depth discussion in the Conclusion section. In response to your comment, we have revised the manuscript to explicitly incorporate application visions related to practical policing scenarios. Specifically, we have added discussions in the revised version to illustrate how the proposed intelligent manipulator can be applied in real-world law enforcement contexts. These include, but are not limited to:

1) Remote handling of hazardous objects, such as suspicious packages or explosive devices, to improve officer safety;
2) Evidence collection in confined or dangerous environments, where human access is limited;
3) Teleoperation in emergency response scenarios, such as disaster rescue or high-risk interventions;
4) Human–robot interaction for tactical assistance, leveraging intuitive gesture-based control to reduce operator training cost and improve responsiveness.

In addition, we surveyed a substantial body of related literature to further support the feasibility of these application directions and to better substantiate the practical significance of the proposed method. We sincerely appreciate your insightful suggestion, which has helped us improve the clarity and practical significance of our work.

“*Furthermore, to enhance the real-world relevance of the proposed system, its potential applications in practical policing scenarios are highlighted. The developed manipulator can be utilized for remote handling of hazardous or suspicious object[43], thereby reducing direct exposure of law enforcement personnel to high-risk environments. In addition, the gesture-driven control framework enables intuitive teleoperation in emergency response tasks, such as disaster rescue and high-risk interventions, improving operational efficiency while lowering the training burden for operators[44,45]. These application visions demonstrate that the proposed system has strong potential for deployment in intelligent policing and public safety domains.*

*Future work will focus on improving recognition robustness under complex environment, reducing system latency, and expanding the gesture vocabulary. Furthermore, more comprehensive comparisons with state-of-the-art methods and real-world deployment in application scenarios such as remote operation and assistive robotics will be investigated[26,46,47].*”

*“[26] Trelinski J, Kwolek B. CNN-based and DTW features for human activity recognition on depth maps[J]. Neural Comput & Applic, 2021, 33:14551–14563*

*[43]	Z Jiang, Y Ma, X Cao, et al. FC-EODR: Immersive Humanoid Dual-Arm Dexterous Explosive Ordnance Disposal Robot[J]. Biomimetics 2023, 8(1):67*

*[44]	W Zhang, H Cheng, L Zhao, et al. A Gesture-Based Teleoperation System for Compliant Robot Motion[J]. Applied Sciences, 2019, 9(24):5290.*

*[45]	J Xie, X Zhen, J Zeng, et al. Human–Robot Interaction Using Dynamic Hand Gesture for Teleoperation of Quadruped Robots with a Robotic Arm[J]. Electronics 2025, 14(5):860.*

*[46]	L X. Human–robot interaction based on gesture and movement recognition[J]. Signal Processing: Image Communication, 2020, 81(115686).*

*[47]	Zick L.A., Martinelli D., Schneider de Oliveira A, et al. Teleoperation system for multiple robots with intuitive hand recognition interface[J]. Scientific Reports, 2024, 14(30230).”*

Reviewer #2:

**Response)** We appreciate the reviewer’s comments. We respond to the reviewer’s comments described below, which will hopefully alleviate the reviewer’s concerns.

(1) Section 2 should be Related Works and not Related work. Section 2.2 is not represented in the proper sentence case.

**Response)** Thank you for pointing this out. We have carefully revised the section titles and formatting throughout the manuscript. Specifically, we changed Section 2 to “Related Works” and corrected the heading of Section 2.2 to follow the proper sentence case style.

(2) The fourth paragraph in section 2 should be checked and corrected appropriately ("o enable accurate gesture recognition while avoiding substantial hardware costs, we adopt a 21-keypoint–based gesture recognition scheme[38], which integrates a CNN based keypoint detection model with a Spatio-Temporal Graph Convolutional Network (ST-GCN).")

**Response)** Thank you for this careful comment. We have thoroughly checked the fourth paragraph in Section 2 and revised the sentence for clarity and grammatical correctness. In particular, we corrected the wording and improved the expression of the description of our 21-keypoint–based gesture recognition scheme, which integrates a CNN-based keypoint detection model with an ST-GCN. We appreciate the reviewer’s attention to detail, which helped us further improve the manuscript.

“*To enable accurate gesture recognition while avoiding substantial hardware costs, we have chosen a 21-keypoint gesture recognition approach[40]—a combined framework utilizing a CNN-based keypoint detection model and a Spatio-Temporal Graph Convolutional Network (ST-GCN).*”

(3) Figure 1 is not well named, it appears too lengthy. Fig 2 is not cited in text. Fig 3 was not named properly.

**Response)** Thank you for these careful and helpful comments. We have revised the figures and their descriptions accordingly. Specifically, we have shortened and refined the caption of Figure 1 to make it more concise and appropriate. We have also added the missing citation of Figure 2 in the main text to ensure proper referencing. In addition, we have corrected the naming and caption of Figure 3 to improve clarity and consistency with the manuscript. These revisions have improved the overall presentation and readability of the figures.

*“****Fig 1. System architecture of the proposed gesture-driven manipulator****”*

*“****Fig 2. Software structure and communication protocol.****”*

*“****Fig 3. Gesture recognition and reproduction results on the manipulator.****”*

(4) The discussion and presentation of the results should be improved as it was not adequately done.

**Response)** Thank you for this valuable comment. We agree that the discussion and presentation of the results needed to be further strengthened. In response, we have substantially revised the Results section to improve both clarity and depth of analysis. Specifically, we have added a more detailed performance analysis in Section 4.3, introduced a comparative study in Section 4.4, and included an ablation study in Section 4.5 to provide a more comprehensive evaluation of the proposed method. We believe these revisions significantly enhance the quality and completeness of the results presentation.

*“As shown in Fig 3, the system successfully reproduces human hand gestures on the manipulator. The average end-to-end response time is approximately 2 seconds, which includes 0.3 seconds for gesture recognition and data preprocessing, 0.7 seconds for wireless communication, and 1.0 second for mechanical execution. The results indicate that the system enables reliable real-time interaction and stable gesture reproduction.”*

*“Traditional methods such as DTW[41] and HMM[42] exhibit lower accuracy and higher latency due to their dependence on handcrafted features and sequential matching. Deep learning-based approaches such as CNN[26] and 3D CNN[27] improve accuracy but require higher computational resources. In contrast, the proposed method achieves a good balance between accuracy (92.6%) and response time (2.0s), while maintaining low hardware cost. This makes it particularly well-suited for embedded and portable applications.”*

*“When keypoint representation is replaced with raw image input (CNN only), the computational cost increases significantly and the response time rises to 2.8 s, making it less suitable for real-time applications. In addition, using a simple classifier instead of ST-GCN results in lower accuracy, demonstrating the effectiveness of graph-based spatial-temporal modeling. Overall, the results confirm that the combination of keypoint detection and ST-GCN provides an optimal trade-off between accuracy, efficiency, and computational cost.”*

(5) The conclusion could be improved and outcome quantified accordingly.

Thank you for this valuable suggestion. We agree that the conclusion can be further strengthened with clearer and more quantitative outcomes. In response, we have carefully revised and rewritten the Conclusion section to better summarize the main findings and to include more explicit quantitative descriptions of the results.

*“This paper presents a lightweight intelligent bionic manipulator system integrating a 15-DOF palm-scale robotic hand with an AI-based gesture-driven control framework. The proposed system achieves a compact and highly integrated design, enabling portable deployment while maintaining dexterous manipulation capability.*

*Experimental results demonstrate that the system achieves an average gesture recognition accuracy of 92.6% and an end-to-end response time of approximately 2.0 s, including 0.3 s for perception, 0.7 s for communication, and 1.0 s for actuation. At the hardware level, all 15 degrees of freedom exhibit stable motion performance, with joint response times ranging from 0.3 s to 1.0 s and rotation angles up to 91°, confirming the effectiveness of the mechanical and control design.*

*Compared with representative methods, the proposed approach provides a favorable trade-off between accuracy, response latency, and hardware cost. Specifically, it reduces response time by up to 28.6% compared with DTW-based methods and maintains competitive accuracy while significantly lowering computational and hardware requirements relative to CNN-based and 3D CNN-based solutions. These results indicate that the proposed system is well-suited for real-time and resource-constrained applications.*

*In addition, the system demonstrates strong robustness and stability in real-time gesture reproduction tasks, enabling reliable human–robot interaction through intuitive vision-based control. The use of embedded hardware (ESP32S3) and commodity smart devices further enhances system scalability and practicality.*

*Future work will focus on improving recognition robustness under complex environment, reducing system latency, and expanding the gesture vocabulary. Furthermore, more comprehensive comparisons with state-of-the-art methods and real-world deployment in application scenarios such as remote operation and assistive robotics will be investigated[26,43,44].”*

(6) This work should be compared with previous related works in the discussion of results section to see how this research performs as compared to the former.

Thank you for this valuable suggestion. We fully agree that a comparison with previous related works would help better demonstrate the effectiveness of the proposed method. In response, we have added a comparative analysis in the Results section (Section 4.4), where our approach is evaluated against several representative methods. This addition provides a clearer view of how the proposed method performs relative to existing work.

**Table 3. Comparison with existing gesture-controlled manipulator systems**

| **Method** | **DOF** | **Recognition Method** | **Accuracy (%)** | **Response Time (s)** | **Hardware Cost** |
| --- | --- | --- | --- | --- | --- |
| DTW-based^[41]^ | 10 | Template matching | 82.5 | 3.5 | Low |
| HMM-based^[42]^ | 12 | Probabilistic model | 85.3 | 3.2 | Medium |
| CNN-base^[26]^ | 15 | 2D CNN | 91.2 | 2.8 | High |
| 3D CNN-base^[27]^ | 15 | 3D CNN | 93.5 | 4.0 | Very High |
| **This work** | **15** | **Keypoint + ST-GCN** | **92.6** | **2.0** | **Low** |

*“ [26]Trelinski J, Kwolek B. CNN-based and DTW features for human activity recognition on depth maps[J]. Neural Comput & Applic, 2021, 33:14551–14563*

*[27]Funke I, Bodenstedt S, Oehme F, et al. Using 3D Convolutional Neural Networks to Learn Spatiotemporal Features for Automatic Surgical Gesture Recognition in Video[J]. MICCAI 2019, 2019:467-475.*

*[41]	Schneider P, Memmesheimer R, Kramer, et al. Gesture Recognition in RGB Videos UsingHuman Body Keypoints and Dynamic Time Warping[J]. RoboCup 2019: Robot World Cup XXIII, 2019: 281–293*

*[42]	Chen F S, Fu C M, Huang C L. Hand gesture recognition using a real-time tracking method and hidden Markov models[J]. Image and Vision Computing, 2003, 21(8):745-758. ”*

(7) There are a number of grammatical and case (capital or small letters) errors. This work could benefit from proofreading.

Thank you for this helpful comment. We acknowledge that there were grammatical and formatting issues in the original manuscript. In response, we have carefully proofread the entire paper and corrected grammatical errors, capitalization inconsistencies, and other language issues to improve clarity and readability. For the reviewer’s convenience, the corresponding revisions have also been reflected in the updated manuscript. We believe these corrections have improved the overall quality of the paper.

(8) What is the novelty or contribution to knowledge?

Thank you for this important question regarding the novelty and contributions of our work. In the revised manuscript, we have significantly strengthened the presentation and clarification of our contributions. Specifically, we have added a clear and explicit contribution summary in the Introduction section, which highlights the main innovations of this study. In addition, we have better positioned our method in relation to existing challenges in the literature to clearly distinguish our approach from prior work. Furthermore, we have reinforced the claimed contributions through comprehensive experimental validation, including comparative experiments and an ablation study, which demonstrate the effectiveness of the proposed method.

*“Although deep learning-based gesture recognition methods, such as convolutional neural networks (CNN[26]) and 3D CNN[27], provide high accuracy, they typically require substantial computational resources, making them less suitable for lightweight and portable systems. On the other hand, traditional methods lack robustness and scalability in dynamic environments. Therefore, there is a strong need for a low-cost, efficient, and real-time gesture-driven control framework that can be seamlessly integrated with high-DOF manipulators.*

*To address these challenges, this paper proposes a lightweight, AI-enhanced intelligent manipulator system that integrates a 15-DOF palm-scale bionic hand with a real-time gesture recognition and control framework. Compared with existing studies, the proposed system emphasizes the balance among system complexity, real-time performance, and hardware cost, making it particularly suitable for embedded and portable applications.*

*The main contributions of this work are summarized as follows:*

*1)	We propose a compact 15-DOF palm-scale bionic manipulator is developed with a highly integrated structure, enabling both dexterity and portability.*

*2)	We introduce a lightweight vision-based gesture recognition framework is designed by combining 21-keypoint detection with a spatio-temporal graph convolutional network, which enables efficient and accurate recognition on resource-constrained devices.*

*3)	We conduct a complete end-to-end system integrating perception, communication, and actuation is implemented, achieving stable real-time control with an average response time of approximately 2 seconds.*

*4)	The system is built using embedded hardware (ESP32S3) and commodity smart devices, providing a practical and scalable solution for real-world applications such as remote manipulation and human–robot interaction.”*

We believe these revisions more clearly and convincingly articulate the novelty and significance of our work.
