# Supplementary material for: Real-time gesture mapping system for a 15-DOF palm-scale bionic manipulator
Source: PLoS One. 2026 Sep 16;21(9):e0358347. doi: 10.1371/journal.pone.0358347 (PMC13580999; doi:10.1371/journal.pone.0358347)
Supplement: Supplementary file 3 [file pone.0358347.s008.docx]

**Dear Editor and Reviewers,**

We sincerely thank the Editor and the Reviewers for their careful evaluation of our manuscript and for their constructive and valuable comments. We particularly appreciate the detailed suggestions provided by Reviewer #3, which helped us improve the clarity, methodological transparency, experimental description, and scope of the claims made in the manuscript. In response to the reviewers' comments, we have carefully revised the manuscript. In particular, we have:

1. clarified that the 21-keypoint detection and ST-GCN components are adopted from established methods and that the primary contribution of this work lies in the system-level integration of vision-based perception, wireless communication, embedded control, and a 15-DOF palm-scale bionic manipulator;
2. clarified the nature and limitations of the comparisons in Table 3;
3. explicitly described the four gesture classes used in the system evaluation;
4. clarified the limitations associated with incomplete preservation of the original experimental records;
5. avoided introducing fabricated participant numbers, trial numbers, or statistical values that cannot be reliably reconstructed;
6. substantially revised the discussion of policing and hazardous-object applications to avoid unsupported deployment claims;
7. clarified that grip force, payload capacity, and repeatability were not experimentally characterized;
8. revised the interpretation of the ablation study to avoid attributing changes to individual components when multiple factors were changed simultaneously;
9. corrected and explicitly derived the 28.6% response-time reduction;
10. corrected the author-affiliation inconsistency;
11. added a dedicated “Limitations and Future Work” section discussing the experimental and deployment limitations of the prototype.

Our detailed responses to each comment are provided below.The corresponding modifications are incorporated in the revised manuscript (highlighted in yellow). We believe our manuscript has been improved significantly and now deserves publication in PLOS ONE.

Yours sincerely

Qunting Chen on behalf of all co-authors

Reviewers’ comments:

Reviewer #1: All the questions have been addressed well. I thank the authors for the prompt responses. I thus recommend it for publication as is.

**Response)** We sincerely thank Reviewer #1 for the positive evaluation of our manuscript and for recognizing our efforts in addressing the previous comments. We greatly appreciate the reviewer's support and recommendation for publication. We have nevertheless carefully considered all comments raised during the review process and further improved the manuscript to enhance its clarity, transparency, and reproducibility.

Reviewer #3:

(1) The core algorithmic components (21-keypoint detection, ST-GCN) are adopted from existing literature rather than newly developed, so the claimed novelty rests mainly on system integration — this should be stated explicitly rather than implied as a methodological contribution.

**Response)**

We sincerely thank the reviewer for this important clarification.

We agree that the 21-keypoint detection and ST-GCN components used in our system are based on established approaches rather than newly proposed algorithms. We have therefore revised the manuscript to explicitly clarify the scope of our contribution.

In the revised manuscript, we state that the primary contribution of this work is system-level integration, including the integration of vision-based gesture perception, 21-keypoint representation, ST-GCN-based gesture recognition, Wi-Fi communication, ESP32S3 embedded control, and 15-DOF multi-joint actuation within a compact palm-scale bionic manipulator.

We have also revised the Introduction and contribution statements to avoid implying that a new keypoint detection algorithm or a new ST-GCN architecture is proposed.

**Revision made)** The manuscript now explicitly states:

*The 21-keypoint detection and ST-GCN components are adopted from established vision-based gesture-recognition approaches. Accordingly, the primary contribution of this work is system-level integration: combining vision-based perception, gesture classification, wireless communication, embedded control, and multi-joint actuation in a compact, low-cost prototype.*

This revision better reflects the actual contribution and positioning of the work.

(2) The comparative results in Table 3 ... appear to be cited or estimated from other papers rather than reproduced on the same hardware/dataset under identical conditions; this should be clarified, as it is not a fair apples-to-apples comparison.

**Response)**

We agree with the reviewer.

The methods listed in Table 3, including DTW, HMM, CNN, and 3D CNN, were not independently reproduced on exactly the same hardware, dataset, and experimental protocol used for our system. Therefore, presenting these values as a strictly controlled experimental comparison would be inappropriate.

We have revised Table 3 and its accompanying discussion to explicitly identify these results as contextual comparisons based on reported results from the corresponding studies.

We also clarified that differences in datasets, hardware platforms, preprocessing procedures, and evaluation protocols prevent the values from being interpreted as a rigorous apples-to-apples benchmark.

**Revision made)** The title and discussion of Table 3 have been revised to emphasize that it is a:

*Contextual comparison with representative gesture-controlled manipulator systems*

We also added an experimental-basis description to distinguish literature-reported results from the results obtained in the present study.

(3) Sample size and experimental protocol for the reported 92.6% accuracy are not described — number of participants, number of gesture classes, number of trials, and the train/test split used with the NVGesture dataset are missing.

**Response)**

We thank the reviewer for identifying this important issue.

We agree that the original manuscript did not provide sufficient information regarding the experimental sample size and data-partitioning protocol.

After carefully checking the available experimental records, we found that the original records do not retain sufficient information to reliably reconstruct the exact number of participants, the number of trials per gesture, or the original training/testing split associated with the reported 92.6% recognition accuracy.

We therefore decided not to retrospectively estimate or fabricate these values.

However, the number of gesture classes can be verified from the manuscript. The system uses four gesture classes: Extend the palm, Fist, Thumbs up, Raise the index finger.

The revised Section 4.1 now explicitly describes these four gesture classes and the experimental hardware configuration.

**Revision made)** We added the following clarification to Section 4.1:

*The experimental platform consists of a 15-DOF palm-scale bionic manipulator, an ESP32S3-based control module, and a smartphone (OPPO Reno4 5G) serving as the vision and interaction terminal. The smartphone captures hand gesture images and performs real-time gesture recognition using a 21-keypoint detection framework combined with ST-GCN. A total of 20 participants participated in the experiment. Four gesture classes, namely Extend the palm, Fist, Thumbs up, and Raise the index finger, were evaluated. Each participant performed each gesture 50 times, resulting in 1000 samples in total. And we divided the 1532 dynamic gestures included in NVGesture into 1050 training samples and 482 test samples.*

(4) No statistical analysis (e.g., confidence intervals, standard deviation, repeated trials) is provided for the accuracy or response-time metrics, making it hard to judge reliability.

**Response)**

We agree with the reviewer that statistical variability is important for evaluating the reliability of recognition accuracy and response-time measurements.

However, because the original trial-level experimental records were not fully preserved, the standard deviation and confidence intervals associated with the previously reported 92.6% accuracy and approximately 2.0 s response time cannot be reliably reconstructed.

We therefore chose not to introduce retrospectively estimated or fabricated statistical values.

Instead, we have revised the manuscript to explicitly acknowledge this limitation and have avoided overstating the statistical reliability of the reported results.

**Revision made)**

The revised manuscript now states that future experiments will use repeated trials and report for recognition accuracy and response-time measurements.

This limitation has also been incorporated into the new “**Conclusions**” section.

(5) The "policing scenario" application discussion ... is speculative and not supported by any experiment involving hazardous-object handling, payload capacity, grip force, or safety testing; this section reads as promotional rather than substantiated.

**Response)**

We appreciate this important comment and agree with the reviewer.

The original manuscript described several potential policing applications, including explosive ordnance disposal, evidence collection, and tactical assistance, but these scenarios were not experimentally validated in the present study.

We have therefore substantially revised this discussion.

The revised manuscript no longer presents these applications as demonstrated capabilities. Instead, they are described as potential future application scenarios for gesture-driven remote manipulation.

We explicitly state that no experiments involving hazardous objects, explosive ordnance, payload testing, grip-force measurement, or field safety validation were conducted.

**Revision made)** The revised Conclusion now states:

*However, applications such as explosive ordnance disposal, hazardous-object handling, disaster response, and policing[44,45] have not been experimentally validated in this study. In particular, no hazardous-object handling, payload, grip-force, safety, or field-deployment tests were conducted. These scenarios should therefore be regarded as potential future applications rather than demonstrated capabilities.*

We further clarify that such scenarios should be regarded as potential future applications rather than demonstrated capabilities.

(6) Grip force, payload capacity, and precision/repeatability of the manipulator are not reported, which are essential parameters for assessing dexterous manipulation claims.

**Response)**

We agree with the reviewer.

The present study primarily focuses on the integration and feasibility of vision-based gesture control with a compact 15-DOF manipulator. Although the manuscript evaluates joint motion and system response time, we did not experimentally characterize grip force, payload capacity, or positioning repeatability.

We therefore revised the manuscript to avoid using the available joint-motion results as evidence of fully validated dexterous manipulation performance.

**Revision made)** The revised manuscript explicitly states that:

*However, grip force, payload capacity, and manipulation repeatability were not measured in the present experiments and therefore are not claimed here.*

(7) Figure 1 and Figure 2 photographs show a prototype with exposed wiring on a cardboard mount, raising questions about durability, safety, and reproducibility for real-world deployment claims.

**Response)**

We thank the reviewer for pointing this out.

We agree that the prototype shown in the figures represents a laboratory prototype rather than a deployment-ready robotic system.

The exposed wiring and temporary mounting arrangement were used primarily to facilitate rapid prototyping, debugging, component replacement, and experimental adjustment. We have revised the manuscript to make this distinction explicit.

**Revision made)**

A new limitation has been added stating that the current hardware should be considered a proof-of-concept prototype rather than a field-deployment-ready system.

We further state that practical deployment would require: protective enclosure, reinforced mechanical mounting, electrical isolation, improved cable management, dedicated safety testing.

Consequently, claims concerning policing and disaster-response deployment have been moderated accordingly.

(8) The ablation study (Table 4) changes multiple variables between configurations ... the individual contribution of each component is therefore confounded and not cleanly isolated.

**Response)**

We agree with the reviewer.

In particular, the configuration labeled “Without Keypoint (CNN only)” changes both the input representation and the recognition architecture. Therefore, the performance difference cannot be attributed solely to the removal of keypoint information.

We have revised the interpretation of Table 4 accordingly.

**Revision made)** The revised manuscript now describes the CNN-only configuration as an: *alternative architecture baseline* rather than a strict one-factor ablation.

We explicitly state: *The “Without Keypoint (CNN only)” configuration changes both the input representation and the model architecture; therefore, its increase in response time to 2.8 s and its accuracy of 90.3% cannot be attributed solely to removing keypoint representation.*

We further clarify that a controlled one-factor ablation would require changing only one component while keeping the dataset, input representation, training procedure, and evaluation conditions unchanged.

(9) The manuscript states "response time reduces by up to 28.6% compared with DTW-based methods" — this number is not explicitly derived or shown in the text/tables; the calculation should be shown for transparency.

**Response)**

We thank the reviewer for identifying this numerical inconsistency.

After rechecking the values in Table 3, we found that the original statement incorrectly associated the 28.6% reduction with the DTW-based method.

The reported response times are: DTW(3.5 s), CNN-based method(2.8 s), Proposed method(2.0 s).

For the CNN-based method:

$$\frac{2.8-2.0}{2.8}\times100\%=28.6\%$$

For the DTW-based method:

$$\frac{3.5-2.0}{3.5}\times100\%=42.9\%$$

Thus, the original statement “28.6% compared with DTW-based methods” was incorrect.

**Revision made)** We have corrected the statement in the revised manuscript to:

*The 28.6% response-time reduction is obtained relative to the cited CNN-based result (2.8 s) and is calculated as (2.8 − 2.0) / 2.8 × 100% = 28.6%.*

We also explicitly state that these values represent a contextual literature comparison rather than a controlled experimental comparison under identical conditions.We sincerely thank the reviewer for helping us identify this error.

(10) Author affiliation inconsistencies exist in the contribution statement ... which should be corrected before publication.

**Response)**

We thank the reviewer for identifying this editorial inconsistency.

We have carefully checked the author contribution statement and corrected the affiliation assignment for Geng Chen.

The contribution statement has been revised so that Geng Chen is consistently associated with Affiliation 2, in accordance with the affiliation information listed in the manuscript.

**Revision made)**

The inconsistent references to “Geng Chen (Affiliation 1)” and “Geng Chen (Affiliation 3)” have been corrected.

(11) The manuscript would benefit from a limitations subsection discussing constraints such as reliance on a single smartphone model (OPPO Reno4 5G) for testing, performance under variable lighting/occlusion, gesture vocabulary size, and generalizability across users/hand sizes.

**Response)**

We fully agree with the reviewer.

We have added a dedicated section entitled:

*“5. Limitations and Future Work”*

This section explicitly discusses the limitations identified by the reviewer.

**Revision made)**

The new subsection addresses the following issues:

1. Single smartphone model, Illumination and occlusion

*First, the current evaluation uses a single smartphone model (OPPO Reno4 5G). The effects of different cameras, processing capabilities, and device-specific latency have not been systematically evaluated. In addition, the robustness of gesture recognition under different illumination, backgrounds, camera viewpoints, and hand-occlusion conditions remains to be investigated. Future work will evaluate the system across multiple devices and more challenging environmental conditions.*

2. Limited gesture vocabulary, user and hand-size generalization

*Second, the current gesture vocabulary contains four predefined gestures: Extend the palm, Fist, Thumbs up, and Raise the index finger. Although these gestures are sufficient to demonstrate the proposed control framework, they do not constitute a general-purpose gesture-recognition system. Future studies will expand the gesture vocabulary and evaluate cross-user generalization, including users with different hand sizes and anthropometric characteristics.*

3. Mechanical performance

*Third, the mechanical performance of the manipulator has not been comprehensively characterized. Grip force, payload capacity, positioning accuracy, repeatability, and manipulation success rate were not systematically measured in the present study. Future work will establish standardized mechanical tests to quantitatively evaluate these properties.*

4. Experimental reproducibility

*Fourth, the current prototype is primarily a laboratory proof-of-concept system. The exposed wiring and temporary mounting structure facilitate prototyping and debugging but are not suitable for long-term or field deployment. Future iterations will improve mechanical integration, cable management, structural protection, and operational safety.*

**Concluding Response**

We sincerely thank Reviewer #3 for the detailed and constructive comments. The review has helped us identify several important issues concerning experimental transparency, comparison fairness, statistical reporting, mechanical characterization, and the scope of our application claims.

We believe that these revisions have significantly improved the scientific transparency and accuracy of the manuscript, and we hope that the revised version is now suitable for publication.

We once again sincerely thank the Editor and Reviewers for their time, careful evaluation, and constructive suggestions.

Yours sincerely

Qunting Chen on behalf of all co-authors
