# Supplementary material for: Real-time gesture mapping system for a 15-DOF palm-scale bionic manipulator
Source: PLoS One. 2026 Sep 16;21(9):e0358347. doi: 10.1371/journal.pone.0358347 (PMC13580999; doi:10.1371/journal.pone.0358347)
Supplement: Supplementary file 7 [file pone.0358347.s005.zip › S5keil-PY32F003A/PY32F002A_Motor_UtoC/MDK-ARM/Output/Project.build_log.htm]

```
# µVision Build Log


## Tool Versions:

IDE-Version: ¦ÌVision V5.38.0.0
Copyright (C) 2022 ARM Ltd and ARM Germany GmbH. All rights reserved.
License Information: mmm Administrator, mmm, LIC=585FJ-UGAQY-DYBV3-A90P3-U4XU1-QUSPJ
 
Tool Versions:
Toolchain:       MDK-ARM Plus  Version: 5.38.0.0
Toolchain Path:  F:\Program Files\Keil_v5\ARM\ARMCLANG\Bin
C Compiler:      ArmClang.exe V6.19
Assembler:       Armasm.exe V6.19
Linker/Locator:  ArmLink.exe V6.19
Library Manager: ArmAr.exe V6.19
Hex Converter:   FromElf.exe V6.19
CPU DLL:         SARMCM3.DLL V5.38.0.0
Dialog DLL:      DARMCM1.DLL V1.19.6.0
Target DLL:      CMSIS_AGDI.dll V1.33.15.0
Dialog DLL:      TARMCM1.DLL V1.14.6.0

## Project:

D:\BaiduNetdiskWorkspace\work\±ÏÉè²ÄÁÏ\ÖÕ¸å5.13\¹¤³ÌÎÄ¼þ\PY32F003A\PY32F002A_Motor_UtoC\MDK-ARM\Project.uvprojx
Project File Date:  02/18/2025

## Output:

*** Using Compiler 'V6.19', folder: 'F:\Program Files\Keil_v5\ARM\ARMCLANG\Bin'
Build target 'PY32F002Ax5_Project'
".\Output\Project.axf" - 0 Error(s), 0 Warning(s).

## Software Packages used:

Package Vendor: ARM
                http://www.keil.com/pack/ARM.CMSIS.5.9.0.pack
                ARM.CMSIS.5.9.0
                CMSIS (Common Microcontroller Software Interface Standard)
   * Component: CORE Version: 5.6.0

Package Vendor: Puya
                https://www.puyasemi.com/uploadfiles/Puya.PY32F0xx_DFP.1.2.0.pack
                Puya.PY32F0xx_DFP.1.2.0
                Puya PY32F0 Series Device Support

## Collection of Component include folders:

./RTE/_PY32F002Ax5_Project
  F:/Program Files/Keil_v5/pack/ARM/CMSIS/5.9.0/CMSIS/Core/Include
  F:/Program Files/Keil_v5/pack/Puya/PY32F0xx_DFP/1.2.0/Drivers/CMSIS/Device/Puya/PY32F0xx/Include

## Collection of Component Files used:

* Component: ARM::CMSIS:CORE:5.6.0
Build Time Elapsed:  00:00:01
```
