# Supplementary material for: Real-time gesture mapping system for a 15-DOF palm-scale bionic manipulator
Source: PLoS One. 2026 Sep 16;21(9):e0358347. doi: 10.1371/journal.pone.0358347 (PMC13580999; doi:10.1371/journal.pone.0358347)
Supplement: Supplementary file 7 [file pone.0358347.s005.zip › S5keil-PY32F003A/PY32F002A_Motor_UtoC/MDK-ARM/Output/Project.htm]

 
  
 Static Call Graph - [.\Output\Project.axf]  
  
 Static Call Graph for image .\Output\Project.axf  
  #&#060CALLGRAPH&#062# ARM Linker, 6190004: Last Updated: Wed Apr  2 20:43:03 2025
  
 Maximum Stack Usage =        168 bytes + Unknown(Cycles, Untraceable Function Pointers)  
Call chain for Maximum Stack Depth: 
main ⇒ $Super$$main ⇒ AT8549_GPIO_Init ⇒ HAL_GPIO_Init
 
 
Mutually Recursive functions
    ADC_COMP_IRQHandler    ⇒    ADC_COMP_IRQHandler  
   HardFault_Handler    ⇒    HardFault_Handler  
 
 
 
Function Pointers
  
   ADC_COMP_IRQHandler  from startup_py32f002xx.o(.text) referenced from startup_py32f002xx.o(RESET)
   EXTI0_1_IRQHandler  from startup_py32f002xx.o(.text) referenced from startup_py32f002xx.o(RESET)
   EXTI2_3_IRQHandler  from startup_py32f002xx.o(.text) referenced from startup_py32f002xx.o(RESET)
   EXTI4_15_IRQHandler  from startup_py32f002xx.o(.text) referenced from startup_py32f002xx.o(RESET)
   FLASH_IRQHandler  from startup_py32f002xx.o(.text) referenced from startup_py32f002xx.o(RESET)
   HardFault_Handler  from py32f0xx_it.o(.text.HardFault_Handler) referenced from startup_py32f002xx.o(RESET)
   I2C1_IRQHandler  from startup_py32f002xx.o(.text) referenced from startup_py32f002xx.o(RESET)
   LPTIM1_IRQHandler  from startup_py32f002xx.o(.text) referenced from startup_py32f002xx.o(RESET)
   NMI_Handler  from py32f0xx_it.o(.text.NMI_Handler) referenced from startup_py32f002xx.o(RESET)
   PendSV_Handler  from py32f0xx_it.o(.text.PendSV_Handler) referenced from startup_py32f002xx.o(RESET)
   RCC_IRQHandler  from startup_py32f002xx.o(.text) referenced from startup_py32f002xx.o(RESET)
   Reset_Handler  from startup_py32f002xx.o(.text) referenced from startup_py32f002xx.o(RESET)
   SPI1_IRQHandler  from startup_py32f002xx.o(.text) referenced from startup_py32f002xx.o(RESET)
   SVC_Handler  from py32f0xx_it.o(.text.SVC_Handler) referenced from startup_py32f002xx.o(RESET)
   SysTick_Handler  from py32f0xx_it.o(.text.SysTick_Handler) referenced from startup_py32f002xx.o(RESET)
   SystemInit  from system_py32f0xx.o(.text.SystemInit) referenced from startup_py32f002xx.o(.text)
   TIM16_IRQHandler  from startup_py32f002xx.o(.text) referenced from startup_py32f002xx.o(RESET)
   TIM1_BRK_UP_TRG_COM_IRQHandler  from startup_py32f002xx.o(.text) referenced from startup_py32f002xx.o(RESET)
   TIM1_CC_IRQHandler  from startup_py32f002xx.o(.text) referenced from startup_py32f002xx.o(RESET)
   USART1_IRQHandler  from py32f0xx_it.o(.text.USART1_IRQHandler) referenced from startup_py32f002xx.o(RESET)
   __main  from entry.o(.ARM.Collect$$$$00000000) referenced from startup_py32f002xx.o(.text)
   fputc  from main.o(.text.fputc) referenced from printf1.o(i.__0printf$1)
   main  from system_py32f0xx.o(.text.$Sub$$main) referenced from entry9a.o(.ARM.Collect$$$$0000000B)
 
 
 
Global Symbols
 
    __main  (Thumb, 0 bytes, Stack size unknown bytes, entry.o(.ARM.Collect$$$$00000000))
 [Address Reference Count : 1]   startup_py32f002xx.o(.text)
 
    _main_stk  (Thumb, 0 bytes, Stack size unknown bytes, entry2.o(.ARM.Collect$$$$00000001))

    _main_scatterload  (Thumb, 0 bytes, Stack size unknown bytes, entry5.o(.ARM.Collect$$$$00000004))
  [Calls]   >>    __scatterload
 

    __main_after_scatterload  (Thumb, 0 bytes, Stack size unknown bytes, entry5.o(.ARM.Collect$$$$00000004))
  [Called By]   >>    __scatterload
 

    _main_clock  (Thumb, 0 bytes, Stack size unknown bytes, entry7b.o(.ARM.Collect$$$$00000008))

    _main_cpp_init  (Thumb, 0 bytes, Stack size unknown bytes, entry8b.o(.ARM.Collect$$$$0000000A))

    _main_init  (Thumb, 0 bytes, Stack size unknown bytes, entry9a.o(.ARM.Collect$$$$0000000B))

    __rt_final_cpp  (Thumb, 0 bytes, Stack size unknown bytes, entry10a.o(.ARM.Collect$$$$0000000D))

    __rt_final_exit  (Thumb, 0 bytes, Stack size unknown bytes, entry11a.o(.ARM.Collect$$$$0000000F))

    Reset_Handler  (Thumb, 8 bytes, Stack size 0 bytes, startup_py32f002xx.o(.text))
 [Address Reference Count : 1]   startup_py32f002xx.o(RESET)
 
    ADC_COMP_IRQHandler  (Thumb, 0 bytes, Stack size 0 bytes, startup_py32f002xx.o(.text))
  [Calls]   >>    ADC_COMP_IRQHandler
 
 [Called By]   >>    ADC_COMP_IRQHandler
 
 [Address Reference Count : 1]   startup_py32f002xx.o(RESET)
 
    EXTI0_1_IRQHandler  (Thumb, 0 bytes, Stack size 0 bytes, startup_py32f002xx.o(.text))
 [Address Reference Count : 1]   startup_py32f002xx.o(RESET)
 
    EXTI2_3_IRQHandler  (Thumb, 0 bytes, Stack size 0 bytes, startup_py32f002xx.o(.text))
 [Address Reference Count : 1]   startup_py32f002xx.o(RESET)
 
    EXTI4_15_IRQHandler  (Thumb, 0 bytes, Stack size 0 bytes, startup_py32f002xx.o(.text))
 [Address Reference Count : 1]   startup_py32f002xx.o(RESET)
 
    FLASH_IRQHandler  (Thumb, 0 bytes, Stack size 0 bytes, startup_py32f002xx.o(.text))
 [Address Reference Count : 1]   startup_py32f002xx.o(RESET)
 
    I2C1_IRQHandler  (Thumb, 0 bytes, Stack size 0 bytes, startup_py32f002xx.o(.text))
 [Address Reference Count : 1]   startup_py32f002xx.o(RESET)
 
    LPTIM1_IRQHandler  (Thumb, 0 bytes, Stack size 0 bytes, startup_py32f002xx.o(.text))
 [Address Reference Count : 1]   startup_py32f002xx.o(RESET)
 
    RCC_IRQHandler  (Thumb, 0 bytes, Stack size 0 bytes, startup_py32f002xx.o(.text))
 [Address Reference Count : 1]   startup_py32f002xx.o(RESET)
 
    SPI1_IRQHandler  (Thumb, 0 bytes, Stack size 0 bytes, startup_py32f002xx.o(.text))
 [Address Reference Count : 1]   startup_py32f002xx.o(RESET)
 
    TIM16_IRQHandler  (Thumb, 0 bytes, Stack size 0 bytes, startup_py32f002xx.o(.text))
 [Address Reference Count : 1]   startup_py32f002xx.o(RESET)
 
    TIM1_BRK_UP_TRG_COM_IRQHandler  (Thumb, 0 bytes, Stack size 0 bytes, startup_py32f002xx.o(.text))
 [Address Reference Count : 1]   startup_py32f002xx.o(RESET)
 
    TIM1_CC_IRQHandler  (Thumb, 0 bytes, Stack size 0 bytes, startup_py32f002xx.o(.text))
 [Address Reference Count : 1]   startup_py32f002xx.o(RESET)
 
    __aeabi_memset  (Thumb, 14 bytes, Stack size 0 bytes, memseta.o(.text), UNUSED)
  [Called By]   >>    _memset$wrapper
  >>    __aeabi_memclr
 

    __aeabi_memset4  (Thumb, 0 bytes, Stack size 0 bytes, memseta.o(.text), UNUSED)

    __aeabi_memset8  (Thumb, 0 bytes, Stack size 0 bytes, memseta.o(.text), UNUSED)

    __aeabi_memclr  (Thumb, 4 bytes, Stack size 0 bytes, memseta.o(.text), UNUSED)
  [Calls]   >>    __aeabi_memset
 

    __aeabi_memclr4  (Thumb, 0 bytes, Stack size 0 bytes, memseta.o(.text))
  [Called By]   >>    HAL_UART_RxCpltCallback
 

    __aeabi_memclr8  (Thumb, 0 bytes, Stack size 0 bytes, memseta.o(.text), UNUSED)

    _memset$wrapper  (Thumb, 18 bytes, Stack size 8 bytes, memseta.o(.text), UNUSED)
  [Calls]   >>    __aeabi_memset
 

    __aeabi_uidiv  (Thumb, 0 bytes, Stack size 16 bytes, uidiv_div0.o(.text))
  [Stack]  Max Depth = 16 Call Chain = __aeabi_uidiv
 
 [Called By]   >>    Step_Motor_Drivers
  >>    UART_SetConfig
  >>    HAL_InitTick
  >>    $Super$$main
 

    __aeabi_uidivmod  (Thumb, 62 bytes, Stack size 16 bytes, uidiv_div0.o(.text), UNUSED)
  [Called By]   >>    _printf_core
 

    __scatterload  (Thumb, 28 bytes, Stack size 0 bytes, init.o(.text))
  [Calls]   >>    __main_after_scatterload
 
 [Called By]   >>    _main_scatterload
 

    __scatterload_rt2  (Thumb, 0 bytes, Stack size 0 bytes, init.o(.text), UNUSED)

    main  (Thumb, 36 bytes, Stack size 8 bytes, system_py32f0xx.o(.text.$Sub$$main))
  [Stack]  Max Depth = 168 Call Chain = main ⇒ $Super$$main ⇒ AT8549_GPIO_Init ⇒ HAL_GPIO_Init
 
 [Calls]   >>    $Super$$main
 
 [Address Reference Count : 1]   entry9a.o(.ARM.Collect$$$$0000000B)
 
    APP_USARTConfig  (Thumb, 172 bytes, Stack size 48 bytes, main.o(.text.APP_USARTConfig))
  [Stack]  Max Depth = 112 Call Chain = APP_USARTConfig ⇒ HAL_UART_Init ⇒ UART_SetConfig ⇒ __aeabi_uidiv
 
 [Calls]   >>    HAL_NVIC_EnableIRQ
  >>    HAL_NVIC_SetPriority
  >>    HAL_GPIO_Init
  >>    HAL_UART_Init
  >>    HAL_UART_DeInit
 
 [Called By]   >>    $Super$$main
 

    AT8549_EN_Vlue  (Thumb, 16 bytes, Stack size 8 bytes, stepper_motor_control.o(.text.AT8549_EN_Vlue))
  [Stack]  Max Depth = 8 Call Chain = AT8549_EN_Vlue
 
 [Calls]   >>    HAL_GPIO_WritePin
 
 [Called By]   >>    $Super$$main
 

    AT8549_GPIO_Init  (Thumb, 140 bytes, Stack size 56 bytes, stepper_motor_control.o(.text.AT8549_GPIO_Init))
  [Stack]  Max Depth = 112 Call Chain = AT8549_GPIO_Init ⇒ HAL_GPIO_Init
 
 [Calls]   >>    HAL_GPIO_WritePin
  >>    HAL_GPIO_Init
 
 [Called By]   >>    $Super$$main
 

    HAL_Delay  (Thumb, 32 bytes, Stack size 16 bytes, py32f0xx_hal.o(.text.HAL_Delay))
  [Stack]  Max Depth = 16 Call Chain = HAL_Delay
 
 [Calls]   >>    HAL_GetTick
 
 [Called By]   >>    Step_Motor_Drivers
 

    HAL_GPIO_Init  (Thumb, 360 bytes, Stack size 56 bytes, py32f0xx_hal_gpio.o(.text.HAL_GPIO_Init))
  [Stack]  Max Depth = 56 Call Chain = HAL_GPIO_Init
 
 [Called By]   >>    AT8549_GPIO_Init
  >>    APP_USARTConfig
 

    HAL_GPIO_WritePin  (Thumb, 16 bytes, Stack size 0 bytes, py32f0xx_hal_gpio.o(.text.HAL_GPIO_WritePin))
  [Called By]   >>    Step_Motor_Drivers
  >>    Step_Motor_Set
  >>    AT8549_EN_Vlue
  >>    AT8549_GPIO_Init
 

    HAL_GetTick  (Thumb, 8 bytes, Stack size 0 bytes, py32f0xx_hal.o(.text.HAL_GetTick))
  [Called By]   >>    HAL_Delay
  >>    HAL_RCC_ClockConfig
  >>    HAL_RCC_OscConfig
 

    HAL_IncTick  (Thumb, 16 bytes, Stack size 0 bytes, py32f0xx_hal.o(.text.HAL_IncTick))
  [Called By]   >>    SysTick_Handler
 

    HAL_Init  (Thumb, 24 bytes, Stack size 8 bytes, py32f0xx_hal.o(.text.HAL_Init))
  [Stack]  Max Depth = 40 Call Chain = HAL_Init ⇒ HAL_InitTick ⇒ __aeabi_uidiv
 
 [Calls]   >>    HAL_InitTick
  >>    HAL_MspInit
 
 [Called By]   >>    $Super$$main
 

    HAL_InitTick  (Thumb, 64 bytes, Stack size 16 bytes, py32f0xx_hal.o(.text.HAL_InitTick))
  [Stack]  Max Depth = 32 Call Chain = HAL_InitTick ⇒ __aeabi_uidiv
 
 [Calls]   >>    HAL_SYSTICK_Config
  >>    HAL_NVIC_SetPriority
  >>    __aeabi_uidiv
 
 [Called By]   >>    HAL_RCC_ClockConfig
  >>    HAL_RCC_OscConfig
  >>    HAL_Init
 

    HAL_MspInit  (Thumb, 40 bytes, Stack size 8 bytes, py32f0xx_hal_msp.o(.text.HAL_MspInit))
  [Stack]  Max Depth = 8 Call Chain = HAL_MspInit
 
 [Called By]   >>    HAL_Init
 

    HAL_NVIC_EnableIRQ  (Thumb, 16 bytes, Stack size 0 bytes, py32f0xx_hal_cortex.o(.text.HAL_NVIC_EnableIRQ))
  [Called By]   >>    APP_USARTConfig
 

    HAL_NVIC_SetPriority  (Thumb, 48 bytes, Stack size 0 bytes, py32f0xx_hal_cortex.o(.text.HAL_NVIC_SetPriority))
  [Called By]   >>    HAL_InitTick
  >>    APP_USARTConfig
 

    HAL_RCC_ClockConfig  (Thumb, 392 bytes, Stack size 32 bytes, py32f0xx_hal_rcc.o(.text.HAL_RCC_ClockConfig))
  [Stack]  Max Depth = 64 Call Chain = HAL_RCC_ClockConfig ⇒ HAL_InitTick ⇒ __aeabi_uidiv
 
 [Calls]   >>    HAL_GetTick
  >>    HAL_InitTick
 
 [Called By]   >>    $Super$$main
 

    HAL_RCC_GetHCLKFreq  (Thumb, 8 bytes, Stack size 0 bytes, py32f0xx_hal_rcc.o(.text.HAL_RCC_GetHCLKFreq))
  [Called By]   >>    Step_Motor_Drivers
 

    HAL_RCC_GetPCLK1Freq  (Thumb, 28 bytes, Stack size 0 bytes, py32f0xx_hal_rcc.o(.text.HAL_RCC_GetPCLK1Freq))
  [Called By]   >>    UART_SetConfig
 

    HAL_RCC_OscConfig  (Thumb, 600 bytes, Stack size 32 bytes, py32f0xx_hal_rcc.o(.text.HAL_RCC_OscConfig))
  [Stack]  Max Depth = 64 Call Chain = HAL_RCC_OscConfig ⇒ HAL_InitTick ⇒ __aeabi_uidiv
 
 [Calls]   >>    HAL_GetTick
  >>    HAL_InitTick
 
 [Called By]   >>    $Super$$main
 

    HAL_SYSTICK_Config  (Thumb, 40 bytes, Stack size 0 bytes, py32f0xx_hal_cortex.o(.text.HAL_SYSTICK_Config))
  [Called By]   >>    Step_Motor_Drivers
  >>    HAL_InitTick
 

    HAL_UART_DeInit  (Thumb, 50 bytes, Stack size 16 bytes, py32f0xx_hal_uart.o(.text.HAL_UART_DeInit))
  [Stack]  Max Depth = 16 Call Chain = HAL_UART_DeInit
 
 [Calls]   >>    HAL_UART_MspDeInit
 
 [Called By]   >>    APP_USARTConfig
 

    HAL_UART_ErrorCallback  (Thumb, 12 bytes, Stack size 8 bytes, main.o(.text.HAL_UART_ErrorCallback))
  [Stack]  Max Depth = 32 Call Chain = HAL_UART_ErrorCallback ⇒ __2printf
 
 [Calls]   >>    __2printf
 
 [Called By]   >>    HAL_UART_IRQHandler
 

    HAL_UART_IRQHandler  (Thumb, 600 bytes, Stack size 48 bytes, py32f0xx_hal_uart.o(.text.HAL_UART_IRQHandler))
  [Stack]  Max Depth = 92 Call Chain = HAL_UART_IRQHandler ⇒ HAL_UART_RxCpltCallback ⇒ HAL_UART_Receive_IT
 
 [Calls]   >>    HAL_UART_TxCpltCallback
  >>    HAL_UART_ErrorCallback
  >>    HAL_UART_RxCpltCallback
 
 [Called By]   >>    USART1_IRQHandler
 

    HAL_UART_Init  (Thumb, 138 bytes, Stack size 24 bytes, py32f0xx_hal_uart.o(.text.HAL_UART_Init))
  [Stack]  Max Depth = 64 Call Chain = HAL_UART_Init ⇒ UART_SetConfig ⇒ __aeabi_uidiv
 
 [Calls]   >>    HAL_UART_MspInit
  >>    UART_SetConfig
 
 [Called By]   >>    APP_USARTConfig
 

    HAL_UART_MspDeInit  (Thumb, 2 bytes, Stack size 0 bytes, py32f0xx_hal_uart.o(.text.HAL_UART_MspDeInit))
  [Called By]   >>    HAL_UART_DeInit
 

    HAL_UART_MspInit  (Thumb, 2 bytes, Stack size 0 bytes, py32f0xx_hal_uart.o(.text.HAL_UART_MspInit))
  [Called By]   >>    HAL_UART_Init
 

    HAL_UART_Receive_IT  (Thumb, 96 bytes, Stack size 28 bytes, py32f0xx_hal_uart.o(.text.HAL_UART_Receive_IT))
  [Stack]  Max Depth = 28 Call Chain = HAL_UART_Receive_IT
 
 [Called By]   >>    HAL_UART_RxCpltCallback
  >>    $Super$$main
 

    HAL_UART_RxCpltCallback  (Thumb, 80 bytes, Stack size 16 bytes, main.o(.text.HAL_UART_RxCpltCallback))
  [Stack]  Max Depth = 44 Call Chain = HAL_UART_RxCpltCallback ⇒ HAL_UART_Receive_IT
 
 [Calls]   >>    __aeabi_memclr4
  >>    HAL_UART_Receive_IT
 
 [Called By]   >>    HAL_UART_IRQHandler
 

    HAL_UART_Transmit_IT  (Thumb, 66 bytes, Stack size 16 bytes, py32f0xx_hal_uart.o(.text.HAL_UART_Transmit_IT))
  [Stack]  Max Depth = 16 Call Chain = HAL_UART_Transmit_IT
 
 [Called By]   >>    fputc
  >>    $Super$$main
 

    HAL_UART_TxCpltCallback  (Thumb, 2 bytes, Stack size 0 bytes, py32f0xx_hal_uart.o(.text.HAL_UART_TxCpltCallback))
  [Called By]   >>    HAL_UART_IRQHandler
 

    HardFault_Handler  (Thumb, 2 bytes, Stack size 0 bytes, py32f0xx_it.o(.text.HardFault_Handler))
  [Calls]   >>    HardFault_Handler
 
 [Called By]   >>    HardFault_Handler
 
 [Address Reference Count : 1]   startup_py32f002xx.o(RESET)
 
    NMI_Handler  (Thumb, 2 bytes, Stack size 0 bytes, py32f0xx_it.o(.text.NMI_Handler))
 [Address Reference Count : 1]   startup_py32f002xx.o(RESET)
 
    PendSV_Handler  (Thumb, 2 bytes, Stack size 0 bytes, py32f0xx_it.o(.text.PendSV_Handler))
 [Address Reference Count : 1]   startup_py32f002xx.o(RESET)
 
    SVC_Handler  (Thumb, 2 bytes, Stack size 0 bytes, py32f0xx_it.o(.text.SVC_Handler))
 [Address Reference Count : 1]   startup_py32f002xx.o(RESET)
 
    Step_Motor_Drivers  (Thumb, 104 bytes, Stack size 24 bytes, stepper_motor_control.o(.text.Step_Motor_Drivers))
  [Stack]  Max Depth = 40 Call Chain = Step_Motor_Drivers ⇒ HAL_Delay
 
 [Calls]   >>    HAL_RCC_GetHCLKFreq
  >>    HAL_GPIO_WritePin
  >>    HAL_Delay
  >>    HAL_SYSTICK_Config
  >>    __aeabi_uidiv
 
 [Called By]   >>    Step_Motor_Set
  >>    Step_Motor_Set0
 

    Step_Motor_Set  (Thumb, 88 bytes, Stack size 24 bytes, stepper_motor_control.o(.text.Step_Motor_Set))
  [Stack]  Max Depth = 64 Call Chain = Step_Motor_Set ⇒ Step_Motor_Drivers ⇒ HAL_Delay
 
 [Calls]   >>    Step_Motor_Drivers
  >>    HAL_GPIO_WritePin
 
 [Called By]   >>    $Super$$main
 

    Step_Motor_Set0  (Thumb, 20 bytes, Stack size 8 bytes, stepper_motor_control.o(.text.Step_Motor_Set0))
  [Stack]  Max Depth = 48 Call Chain = Step_Motor_Set0 ⇒ Step_Motor_Drivers ⇒ HAL_Delay
 
 [Calls]   >>    Step_Motor_Drivers
 
 [Called By]   >>    $Super$$main
 

    SysTick_Handler  (Thumb, 8 bytes, Stack size 8 bytes, py32f0xx_it.o(.text.SysTick_Handler))
  [Stack]  Max Depth = 8 Call Chain = SysTick_Handler
 
 [Calls]   >>    HAL_IncTick
 
 [Address Reference Count : 1]   startup_py32f002xx.o(RESET)
 
    SystemInit  (Thumb, 32 bytes, Stack size 0 bytes, system_py32f0xx.o(.text.SystemInit))
 [Address Reference Count : 1]   startup_py32f002xx.o(.text)
 
    USART1_IRQHandler  (Thumb, 12 bytes, Stack size 8 bytes, py32f0xx_it.o(.text.USART1_IRQHandler))
  [Stack]  Max Depth = 100 Call Chain = USART1_IRQHandler ⇒ HAL_UART_IRQHandler ⇒ HAL_UART_RxCpltCallback ⇒ HAL_UART_Receive_IT
 
 [Calls]   >>    HAL_UART_IRQHandler
 
 [Address Reference Count : 1]   startup_py32f002xx.o(RESET)
 
    fputc  (Thumb, 24 bytes, Stack size 16 bytes, main.o(.text.fputc))
  [Stack]  Max Depth = 32 Call Chain = fputc ⇒ HAL_UART_Transmit_IT
 
 [Calls]   >>    HAL_UART_Transmit_IT
 
 [Address Reference Count : 1]   printf1.o(i.__0printf$1)
 
    $Super$$main  (Thumb, 232 bytes, Stack size 48 bytes, main.o(.text.main))
  [Stack]  Max Depth = 160 Call Chain = $Super$$main ⇒ AT8549_GPIO_Init ⇒ HAL_GPIO_Init
 
 [Calls]   >>    HAL_UART_Transmit_IT
  >>    Step_Motor_Set
  >>    Step_Motor_Set0
  >>    AT8549_EN_Vlue
  >>    AT8549_GPIO_Init
  >>    HAL_UART_Receive_IT
  >>    APP_USARTConfig
  >>    HAL_RCC_ClockConfig
  >>    HAL_RCC_OscConfig
  >>    HAL_Init
  >>    __aeabi_uidiv
 
 [Called By]   >>    main
 

    __0printf$1  (Thumb, 24 bytes, Stack size 24 bytes, printf1.o(i.__0printf$1), UNUSED)
  [Calls]   >>    _printf_core
 

    __1printf$1  (Thumb, 0 bytes, Stack size 24 bytes, printf1.o(i.__0printf$1), UNUSED)

    __2printf  (Thumb, 0 bytes, Stack size 24 bytes, printf1.o(i.__0printf$1))
  [Stack]  Max Depth = 24 Call Chain = __2printf
 
 [Called By]   >>    HAL_UART_ErrorCallback
 

    __scatterload_copy  (Thumb, 14 bytes, Stack size unknown bytes, handlers.o(i.__scatterload_copy), UNUSED)

    __scatterload_null  (Thumb, 2 bytes, Stack size unknown bytes, handlers.o(i.__scatterload_null), UNUSED)

    __scatterload_zeroinit  (Thumb, 14 bytes, Stack size unknown bytes, handlers.o(i.__scatterload_zeroinit), UNUSED)
 
 
Local Symbols
 
    UART_SetConfig  (Thumb, 196 bytes, Stack size 24 bytes, py32f0xx_hal_uart.o(.text.UART_SetConfig))
  [Stack]  Max Depth = 40 Call Chain = UART_SetConfig ⇒ __aeabi_uidiv
 
 [Calls]   >>    HAL_RCC_GetPCLK1Freq
  >>    __aeabi_uidiv
 
 [Called By]   >>    HAL_UART_Init
 

    _printf_core  (Thumb, 332 bytes, Stack size 88 bytes, printf1.o(i._printf_core), UNUSED)
  [Calls]   >>    __aeabi_uidivmod
 
 [Called By]   >>    __0printf$1
 
 
 
Undefined Global Symbols
