# Supplementary material for: Real-time gesture mapping system for a 15-DOF palm-scale bionic manipulator
Source: PLoS One. 2026 Sep 16;21(9):e0358347. doi: 10.1371/journal.pone.0358347 (PMC13580999; doi:10.1371/journal.pone.0358347)
Supplement: Peer review history

# Peer review: Decision Letter 0

**DOI:** 10.1371/journal.pone.0358347.r001

**Authors:** Andrea Tigrini

25 Feb 2026

Dear Dr. Chen,

Thank you for submitting your manuscript to PLOS ONE. After careful consideration, we feel that it has merit but does not fully meet PLOS ONE’s publication criteria as it currently stands. Therefore, we invite you to submit a revised version of the manuscript that addresses the points raised during the review process.

==============================

**ACADEMIC EDITOR:**

Thank you for submitting your manuscript to PLOS ONE. After a thorough review by two experts in the field, I am inviting you to revise the manuscript to address the significant concerns raised.

While both reviewers find the 15-DOF palm-scale bionic manipulator system interesting and technically promising, they have identified critical gaps that must be addressed before the paper can be considered for publication. Specifically, you should focus your revision on the following  pillars:

1)Both reviewers noted that the specific contributions of this work are currently unclear. You must explicitly "distill" your innovations (as suggested by Reviewer 1) by identifying the fundamental bottlenecks in gesture mapping and explaining how your 15-DOF system provides a targeted solution that differs from existing literature.

2)A major weakness identified is the lack of comparative experiments. To demonstrate the effectiveness of your proposed method.

3) Please provide a vision for how this work connects to practical scenarios (e.g., hospital monitoring or policing) to enhance its real-world relevance. Ensure you have integrated recent, relevant literature (including works on facial/emotion classification and gesture frameworks) to embed your work within the current state of the art.

Please include a point-by-point response to all reviewer comments and a tracked-changes version of your manuscript.

Best regards,

Andrea Tigrini

Academic Editor

PLOS ONE

==============================

Please submit your revised manuscript by Apr 11 2026 11:59PM. If you will need more time than this to complete your revisions, please reply to this message or contact the journal office at plosone@plos.org. When you're ready to submit your revision, log on to https://www.editorialmanager.com/pone/ and select the 'Submissions Needing Revision' folder to locate your manuscript file.

- A letter that responds to each point raised by the academic editor and reviewer(s). You should upload this letter as a separate file labeled 'Response to Reviewers'.
- A marked-up copy of your manuscript that highlights changes made to the original version. You should upload this as a separate file labeled 'Revised Manuscript with Track Changes'.
- An unmarked version of your revised paper without tracked changes. You should upload this as a separate file labeled 'Manuscript'.

If you would like to make changes to your financial disclosure, please include your updated statement in your cover letter. Guidelines for resubmitting your figure files are available below the reviewer comments at the end of this letter.

If applicable, we recommend that you deposit your laboratory protocols in protocols.io to enhance the reproducibility of your results. Protocols.io assigns your protocol its own identifier (DOI) so that it can be cited independently in the future. For instructions see: https://journals.plos.org/plosone/s/submission-guidelines#loc-laboratory-protocols. Additionally, PLOS ONE offers an option for publishing peer-reviewed Lab Protocol articles, which describe protocols hosted on protocols.io. Read more information on sharing protocols at https://plos.org/protocols?utm_medium=editorial-email&utm_source=authorletters&utm_campaign=protocols.

We look forward to receiving your revised manuscript.

Kind regards,

Andrea Tigrini, Ph.D.

Academic Editor

PLOS One

Journal requirements:

When submitting your revision, we need you to address these additional requirements.

1. Please ensure that your manuscript meets PLOS ONE's style requirements, including those for file naming. The PLOS ONE style templates can be found at

https://journals.plos.org/plosone/s/file?id=wjVg/PLOSOne_formatting_sample_main_body.pdf and

https://journals.plos.org/plosone/s/file?id=ba62/PLOSOne_formatting_sample_title_authors_affiliations.pdf

2. Please note that PLOS One has specific guidelines on code sharing for submissions in which author-generated code underpins the findings in the manuscript. In these cases, we expect all author-generated code to be made available without restrictions upon publication of the work. Please review our guidelines at https://journals.plos.org/plosone/s/materials-and-software-sharing#loc-sharing-code and ensure that your code is shared in a way that follows best practice and facilitates reproducibility and reuse.

3. Please ensure that you include a title page within your main document. We do appreciate that you have a title page document uploaded as a separate file, however, as per our author guidelines (http://journals.plos.org/plosone/s/submission-guidelines#loc-title-page) we do require this to be part of the manuscript file itself and not uploaded separately.

Could you therefore please include the title page into the beginning of your manuscript file itself, listing all authors and affiliations.

4. We note that Figures 1, 2 and 3 in your submission contain copyrighted images. All PLOS content is published under the Creative Commons Attribution License (CC BY 4.0), which means that the manuscript, images, and Supporting Information files will be freely available online, and any third party is permitted to access, download, copy, distribute, and use these materials in any way, even commercially, with proper attribution. For more information, see our copyright guidelines: http://journals.plos.org/plosone/s/licenses-and-copyright.

We require you to either (1) present written permission from the copyright holder to publish these figures specifically under the CC BY 4.0 license, or (2) remove the figures from your submission:

1. You may seek permission from the original copyright holder of Figures 1, 2 and 3 to publish the content specifically under the CC BY 4.0 license.

We recommend that you contact the original copyright holder with the Content Permission Form (http://journals.plos.org/plosone/s/file?id=7c09/content-permission-form.pdf) and the following text:

“I request permission for the open-access journal PLOS ONE to publish XXX under the Creative Commons Attribution License (CCAL) CC BY 4.0 (http://creativecommons.org/licenses/by/4.0/). Please be aware that this license allows unrestricted use and distribution, even commercially, by third parties. Please reply and provide explicit written permission to publish XXX under a CC BY license and complete the attached form.”

Please upload the completed Content Permission Form or other proof of granted permissions as an "Other" file with your submission.

In the figure caption of the copyrighted figure, please include the following text: “Reprinted from [ref] under a CC BY license, with permission from [name of publisher], original copyright [original copyright year].”

2. If you are unable to obtain permission from the original copyright holder to publish these figures under the CC BY 4.0 license or if the copyright holder’s requirements are incompatible with the CC BY 4.0 license, please either i) remove the figure or ii) supply a replacement figure that complies with the CC BY 4.0 license. Please check copyright information on all replacement figures and update the figure caption with source information. If applicable, please specify in the figure caption text when a figure is similar but not identical to the original image and is therefore for illustrative purposes only.

5. Please upload a copy of Figure 6, to which you refer in your text on page 8. If the figure is no longer to be included as part of the submission please remove all reference to it within the text.

6. Please include captions for your Supporting Information files at the end of your manuscript, and update any in-text citations to match accordingly. Please see our Supporting Information guidelines for more information: http://journals.plos.org/plosone/s/supporting-information.

If the reviewer comments include a recommendation to cite specific previously published works, please review and evaluate these publications to determine whether they are relevant and should be cited. There is no requirement to cite these works unless the editor has indicated otherwise.

Additional Editor Comments:

Thank you for submitting your manuscript to PLOS ONE. After a thorough review by two experts in the field, I am inviting you to revise the manuscript to address the significant concerns raised.

While both reviewers find the 15-DOF palm-scale bionic manipulator system interesting and technically promising, they have identified critical gaps that must be addressed before the paper can be considered for publication. Specifically, you should focus your revision on the following pillars:

1)Both reviewers noted that the specific contributions of this work are currently unclear. You must explicitly "distill" your innovations (as suggested by Reviewer 1) by identifying the fundamental bottlenecks in gesture mapping and explaining how your 15-DOF system provides a targeted solution that differs from existing literature.

2)A major weakness identified is the lack of comparative experiments. To demonstrate the effectiveness of your proposed method.

3) Please provide a vision for how this work connects to practical scenarios (e.g., hospital monitoring or policing) to enhance its real-world relevance. Ensure you have integrated recent, relevant literature (including works on facial/emotion classification and gesture frameworks) to embed your work within the current state of the art.

Please include a point-by-point response to all reviewer comments and a tracked-changes version of your manuscript.

[Note: HTML markup is below. Please do not edit.]

Reviewers' comments:

Reviewer's Responses to Questions

**Comments to the Author**

1. Is the manuscript technically sound, and do the data support the conclusions?

Reviewer #1: Yes

Reviewer #2: Partly

**********

2. Has the statistical analysis been performed appropriately and rigorously?

Reviewer #1: Yes

Reviewer #2: No

**********

3. Have the authors made all data underlying the findings in their manuscript fully available?

The PLOS Data policy

Reviewer #1: Yes

Reviewer #2: Yes

**********

4. Is the manuscript presented in an intelligible fashion and written in standard English?

Reviewer #1: Yes

Reviewer #2: Yes

**********

Reviewer #1: This paper presents a gesture mapping system for a 15-DOF palm-scale bionic manipulator. The topic is interesting, and the paper’s topic is also highly aligned with the academic programs of this institution. While some key issues should be further clarified below:

Q1: The paper’s innovations should be more clearly distilled, ideally by grounding them in the fundamental bottlenecks of the field and formulating targeted solutions accordingly.

Q2：Although the techniques in this paper are practical and relatively mature, the authors are encouraged to cite more relevant literature (FE-SpikeFormer: A Camera-based Facial Expression Recognition method for Hospital Health Monitoring； A Dual-Pathway Driver Emotion Classification Network Using Multi-Task Learning Strategy: A Joint Verification) to demonstrate their effectiveness and adoption in real-world applications.

Q3：The major weakness of the paper lies in the insufficient comparative experiments. The authors are encouraged to add more comprehensive comparisons; doing so would better highlight the strengths of the proposed method and significantly improve the completeness of the work.

Q4：It is recommended that the paper provide some application visions that connect the proposed work with practical policing scenarios, in order to enhance its real-world relevance.

Reviewer #2: Section 2 should be Related Works and not Related work

Section 2.2 is not represented in the proper sentence case

The fourth paragraph in section 2 should be checked and corrected appropriately ("o enable accurate gesture recognition while avoiding substantial hardware costs, we adopt a 21-keypoint–based gesture recognition scheme[38], which integrates a CNN based keypoint detection model with a Spatio-Temporal Graph Convolutional Network (ST-GCN).")

figure 1 is not well named, it appears too lengthy.

Fig 2 is not cited in text

Fig 3 was not named properly

The discussion and presentation of the results should be improved as it was not adequately done.

The conclusion could be improved and outcome quantified accordingly.

This work should be compared with previous related works in the discussion of results section to see how this research performs as compared to the former.

There are a number of grammatical and case (capital or small letters) errors. This work could benefit from proofreading.

what is the novelty or contribution to knowledge?

**********

what does this mean?). If published, this will include your full peer review and any attached files.

If you choose “no”, your identity will remain anonymous but your review may still be made public.

**Do you want your identity to be public for this peer review?**  For information about this choice, including consent withdrawal, please see our Privacy Policy

Reviewer #1: No

Reviewer #2: **Yes:** NYANGWARIMAM OBADIAH ALI

**********

[NOTE: If reviewer comments were submitted as an attachment file, they will be attached to this email and accessible via the submission site. Please log into your account, locate the manuscript record, and check for the action link "View Attachments". If this link does not appear, there are no attachment files.]

To ensure your figures meet our technical requirements, please review our figure guidelines: https://journals.plos.org/plosone/s/figures

You may also use PLOS’s free figure tool, NAAS, to help you prepare publication quality figures: https://journals.plos.org/plosone/s/figures#loc-tools-for-figure-preparation.

NAAS will assess whether your figures meet our technical requirements by comparing each figure against our figure specifications.

# Author comment: Author response to Decision Letter 1

**DOI:** 10.1371/journal.pone.0358347.r002

5 May 2026

Dear Reviewer,

We appreciate reviewer’s comments and valuable queries, which have helped us improve the clarity and quality of our manuscript. Given below are detailed point-by-point responses to the questions and suggestions. The corresponding modifications are incorporated in the revised manuscript (highlighted in yellow). We believe our manuscript has been improved significantly and now deserves publication in PLOS ONE.

Yours sincerely

Xinhui Chen on behalf of all co-authors

Reviewers’ comments:

Reviewer #1: This paper presents a gesture mapping system for a 15-DOF palm-scale bionic manipulator. The topic is interesting, and the paper’s topic is also highly aligned with the academic programs of this institution. While some key issues should be further clarified below:

Response) We appreciate the reviewer’s comments “The topic is interesting”. We respond to the reviewer’s comments described below, which will hopefully alleviate the reviewer’s concerns.

(1) The paper’s innovations should be more clearly distilled, ideally by grounding them in the fundamental bottlenecks of the field and formulating targeted solutions accordingly.

Response) We appreciate the reviewer for this important question. In the revised manuscript, we clearly identified the key challenges in high-DOF manipulator control in page 2-3.

These revisions significantly improve the clarity and emphasis of the paper’s novelty.

“Although deep learning-based gesture recognition methods, such as convolutional neural networks (CNN[26]) and 3D CNN[27], provide high accuracy, they typically require substantial computational resources, making them less suitable for lightweight and portable systems. On the other hand, traditional methods lack robustness and scalability in dynamic environments. Therefore, there is a strong need for a low-cost, efficient, and real-time gesture-driven control framework that can be seamlessly integrated with high-DOF manipulators.

To address these challenges, this paper proposes a lightweight, AI-enhanced intelligent manipulator system that integrates a 15-DOF palm-scale bionic hand with a real-time gesture recognition and control framework. Compared with existing studies, the proposed system emphasizes the balance among system complexity, real-time performance, and hardware cost, making it particularly suitable for embedded and portable applications.

The main contributions of this work are summarized as follows:

1) We propose a compact 15-DOF palm-scale bionic manipulator is developed with a highly integrated structure, enabling both dexterity and portability.

2) We introduce a lightweight vision-based gesture recognition framework is designed by combining 21-keypoint detection with a spatio-temporal graph convolutional network, which enables efficient and accurate recognition on resource-constrained devices.

3) We conduct a complete end-to-end system integrating perception, communication, and actuation is implemented, achieving stable real-time control with an average response time of approximately 2 seconds.

4) The system is built using embedded hardware (ESP32S3) and commodity smart devices, providing a practical and scalable solution for real-world applications such as remote manipulation and human–robot interaction.”

(2) Although the techniques in this paper are practical and relatively mature, the authors are encouraged to cite more relevant literature (FE-SpikeFormer: A Camera-based Facial Expression Recognition method for Hospital Health Monitoring； A Dual-Pathway Driver Emotion Classification Network Using Multi-Task Learning Strategy: A Joint Verification) to demonstrate their effectiveness and adoption in real-world applications.

Response) We feel sorry that the number of relevant references does not meet the requirements. We list some important relevant literature as below.

“[38] Dong Z, Zhu L, Zhou S, et al. FE-SpikeFormer: A Camera-Based Facial Expression Recognition Method for Hospital Health Monitoring[J]. IEEE Journal of Biomedical and Health Informatics, 2025:1-11.

[39] Z. Dong, C. Hu, L. Zhu, et al. A Dual-Pathway Driver Emotion Classification Network Using Multitask Learning Strategy: A Joint Verification[J]. IEEE Internet of Things Journal, 2025, 12(10):14897-14908.”

In particular, we have incorporated references to FE-SpikeFormer, a camera-based recognition system, and dual-pathway emotion classification networks. These works are discussed in Section 2.2 to highlight the practical effectiveness of deep learning-based perception systems in real-world applications.

“Recent studies have demonstrated the effectiveness of deep learning-based perception systems in real-world applications. For example, FE-SpikeFormer introduces a camera-based facial expression recognition framework for healthcare monitorin[38], while dual-pathway emotion recognition networks further validate the robustness of multi-task learning strategies in dynamic environments[39]. These works highlight the practicality and scalability of deep learning-based recognition systems.”

(3) The major weakness of the paper lies in the insufficient comparative experiments. The authors are encouraged to add more comprehensive comparisons; doing so would better highlight the strengths of the proposed method and significantly improve the completeness of the work.

Response) We sincerely appreciate this important comment. In response, we have strengthened the experimental validation in several ways. First, we added a new comparative experiment in Table 3, where our method is evaluated against DTW, HMM, CNN, and 3D CNN baselines. Second, we introduced a new discussion subsection in Section 4.4 to analyze the performance differences in greater detail. Third, we included quantitative metrics such as accuracy, response time, and hardware cost to provide a more comprehensive evaluation.

Table 3. Comparison with existing gesture-controlled manipulator systems

In addition, we further enhanced the experimental section by adding an ablation study in Table 4 to assess the contributions of the key components, including ST-GCN and the keypoint representation. These revisions substantially improve the completeness and rigor of the experimental validation.

Table 4. Ablation study of the proposed method

(4) It is recommended that the paper provide some application visions that connect the proposed work with practical policing scenarios, in order to enhance its real-world relevance.

Response) Thank you very much for this insightful and valuable suggestion. We fully agree that connecting the proposed work with practical policing scenarios would further enhance its real-world relevance. Although we were unable to carry out additional application-specific experiments due to the current scope and limitations of our study, we have made our best effort to enrich the manuscript by adding a more in-depth discussion in the Conclusion section. In response to your comment, we have revised the manuscript to explicitly incorporate application visions related to practical policing scenarios. Specifically, we have added discussions in the revised version to illustrate how the proposed intelligent manipulator can be applied in real-world law enforcement contexts. These include, but are not limited to:

1) Remote handling of hazardous objects, such as suspicious packages or explosive devices, to improve officer safety;

2) Evidence collection in confined or dangerous environments, where human access is limited;

3) Teleoperation in emergency response scenarios, such as disaster rescue or high-risk interventions;

4) Human–robot interaction for tactical assistance, leveraging intuitive gesture-based control to reduce operator training cost and improve responsiveness.

In addition, we surveyed a substantial body of related literature to further support the feasibility of these application directions and to better substantiate the practical significance of the proposed method. We sincerely appreciate your insightful suggestion, which has helped us improve the clarity and practical significance of our work.

“Furthermore, to enhance the real-world relevance of the proposed system, its potential applications in practical policing scenarios are highlighted. The developed manipulator can be utilized for remote handling of hazardous or suspicious object[43], thereby reducing direct exposure of law enforcement personnel to high-risk environments. In addition, the gesture-driven control framework enables intuitive teleoperation in emergency response tasks, such as disaster rescue and high-risk interventions, improving operational efficiency while lowering the training burden for operators[44,45]. These application visions demonstrate that the proposed system has strong potential for deployment in intelligent policing and public safety domains.

Future work will focus on improving recognition robustness under complex environment, reducing system latency, and expanding the gesture vocabulary. Furthermore, more comprehensive comparisons with state-of-the-art methods and real-world deployment in application scenarios such as remote operation and assistive robotics will be investigated[26,46,47].”

“[26] Trelinski J, Kwolek B. CNN-based and DTW features for human activity recognition on depth maps[J]. Neural Comput & Applic, 2021, 33:14551–14563

[43] Z Jiang, Y Ma, X Cao, et al. FC-EODR: Immersive Humanoid Dual-Arm Dexterous Explosive Ordnance Disposal Robot[J]. Biomimetics 2023, 8(1):67

[44] W Zhang, H Cheng, L Zhao, et al. A Gesture-Based Teleoperation System for Compliant Robot Motion[J]. Applied Sciences, 2019, 9(24):5290.

[45] J Xie, X Zhen, J Zeng, et al. Human–Robot Interaction Using Dynamic Hand Gesture for Teleoperation of Quadruped Robots with a Robotic Arm[J]. Electronics 2025, 14(5):860.

[46] L X. Human–robot interaction based on gesture and movement recognition[J]. Signal Processing: Image Communication, 2020, 81(115686).

[47] Zick L.A., Martinelli D., Schneider de Oliveira A, et al. Teleoperation system for multiple robots with intuitive hand recognition interface[J]. Scientific Reports, 2024, 14(30230).”

Reviewer #2:

Response) We appreciate the reviewer’s comments. We respond to the reviewer’s comments described below, which will hopefully alleviate the reviewer’s concerns.

(1) Section 2 should be Related Works and not Related work. Section 2.2 is not represented in the proper sentence case.

Response) Thank you for pointing this out. We have carefully revised the section titles and formatting throughout the manuscript. Specifically, we changed Section 2 to “Related Works” and corrected the heading of Section 2.2 to follow the proper sentence case style.

(2) The fourth paragraph in section 2 should be checked and corrected appropriately ("o enable accurate gesture recognition while avoiding substantial hardware costs, we adopt a 21-keypoint–based gesture recognition scheme[38], which integrates a CNN based keypoint detection model with a Spatio-Temporal Graph Convolutional Network (ST-GCN).")

Response) Thank you for this careful comment. We have thoroughly checked the fourth paragraph in Section 2 and revised the sentence for clarity and grammatical correctness. In particular, we corrected the wording and improved the expression of the description of our 21-keypoint–based gesture recognition scheme, which integrates a CNN-based keypoint detection model with an ST-GCN. We appreciate the reviewer’s attention to detail, which helped us further improve the manuscript.

“To enable accurate gesture recognition while avoiding substantial hardware costs, we have chosen a 21-keypoint gesture recognition approach[40]—a combined framework utilizing a CNN-based keypoint detection model and a Spatio-Temporal Graph Convolutional Network (ST-GCN).”

(3) Figure 1 is not well named, it appears too lengthy. Fig 2 is not cited in text. Fig 3 was not named properly.

Response) Thank you for these careful and helpful comments. We have revised the figures and their descriptions accordingly. Specifically, we have shortened and refined the caption of Figure 1 to make it more concise and appropriate. We have also added the missing citation of Figure 2 in the main text to ensure proper referencing. In addition, we have corrected the naming and caption of Figure 3 to improve clarity and consistency with the manuscript. These revisions have improved the overall presentation and readability of the figures.

“Fig 1. System architecture of the proposed gesture-driven manipulator”

“Fig 2. Software structure and communication protocol.”

“Fig 3. Gesture recognition and reproduction results on the manipulator.”

(4) The discussion and presentation of the results should be improved as it was not adequately done.

Response) Thank you for this valuable comment. We agree that the discussion and presentation of the results needed to be further strengthened. In response, we have substantially revised the Results section to improve both clarity and depth of analysis. Specifically, we have added a more detailed performance analysis in Section 4.3, introduced a comparative study in Section 4.4, and included an ablation study in Section 4.5 to provide a more comprehensive evaluation of the proposed method. We believe these revisions significantly enhance the quality and completeness of the results presentation.

“As shown in Fig 3, the system successfully reproduces human hand gestures on the manipulator. The average end-to-end response time is approximately 2 seconds, which includes 0.3 seconds for gesture recognition and data preprocessing, 0.7 seconds for wireless communication, and 1.0 second for mechanical execution. The results indicate that the system enables reliable real-time interaction and stable gesture reproduction.”

“Traditional methods such as DTW[41] and HMM[42] exhibit lower accuracy and higher latency due to their dependence on handcrafted features and sequential matching. Deep learning-based approaches such as CNN[26] and 3D CNN[27] improve accuracy but require higher computational resources. In contrast, the proposed method achieves a good balance between accuracy (92.6%) and response time (2.0s), while maintaining low hardware cost. This makes it particularly well-suited for embedded and portable applications.”

“When keypoint representation is replaced with raw image input (CNN only), the computational cost increases significantly and the response time rises to 2.8 s, making it less suitable for real-time applications. In addition, using a simple classifier instead of ST-GCN results in lower accuracy, demonstrating the effectiveness of graph-based spatial-temporal modeling. Overall, the results confirm that the combination of keypoint detection and ST-GCN provides an optimal trade-off between accuracy, efficiency, and computational cost.”

(5) The conclusion could be improved and outcome quantified accordingly.

Thank you for this valuable suggestion. We agree that the conclusion can be further strengthened with clearer and more quantitative outcomes. In response, we have carefully revised and rewritten the Conclusion section to better summarize the main findings and to include more explicit quantitative descriptions of the results.

“This paper presents a lightweight intelligent bionic manipulator system integrating a 15-DOF palm-scale robotic hand with an AI-based gesture-driven control framework. The proposed system achieves a compact and highly integrated design, enabling portable deployment while maintaining dexterous manipulation capability.

Experimental results demonstrate that the system achieves an average gesture recognition accuracy of 92.6% and an end-to-end response time of approximately 2.0 s, including 0.3 s for perception, 0.7 s for communication, and 1.0 s for actuation. At the hardware level, all 15 degrees of freedom exhibit stable motion performance, with joint response times ranging from 0.3 s to 1.0 s and rotation angles up to 91°, confirming the effectiveness of the mechanical and control design.

Compared with representative methods, the proposed approach provides a favorable trade-off between accuracy, response latency, and hardware cost. Specifically, it reduces response time by up to 28.6% compared with DTW-based methods and maintains competitive accuracy while significantly

Attachment Submitted filename: Response to Reviewers.docx

# Peer review: Decision Letter 1

**DOI:** 10.1371/journal.pone.0358347.r003

**Authors:** Usman Ghafoor

12 Aug 2026

Dear Dr. Chen,

Thank you for submitting your manuscript to PLOS ONE. After careful consideration, we feel that it has merit but does not fully meet PLOS ONE’s publication criteria as it currently stands. Therefore, we invite you to submit a revised version of the manuscript that addresses the points raised during the review process.

Please submit your revised manuscript by Sep 26 2026 11:59PM. If you will need more time than this to complete your revisions, please reply to this message or contact the journal office at plosone@plos.org. When you're ready to submit your revision, log on to https://www.editorialmanager.com/pone/ and select the 'Submissions Needing Revision' folder to locate your manuscript file.

- A letter that responds to each point raised by the academic editor and reviewer(s). You should upload this letter as a separate file labeled 'Response to Reviewers'.
- A marked-up copy of your manuscript that highlights changes made to the original version. You should upload this as a separate file labeled 'Revised Manuscript with Track Changes'.
- An unmarked version of your revised paper without tracked changes. You should upload this as a separate file labeled 'Manuscript'.

If you would like to make changes to your financial disclosure, please include your updated statement in your cover letter. Guidelines for resubmitting your figure files are available below the reviewer comments at the end of this letter.

If applicable, we recommend that you deposit your laboratory protocols in protocols.io to enhance the reproducibility of your results. Protocols.io assigns your protocol its own identifier (DOI) so that it can be cited independently in the future. For instructions see: https://journals.plos.org/plosone/s/submission-guidelines#loc-laboratory-protocols. Additionally, PLOS ONE offers an option for publishing peer-reviewed Lab Protocol articles, which describe protocols hosted on protocols.io. Read more information on sharing protocols at https://plos.org/protocols?utm_medium=editorial-email&utm_source=authorletters&utm_campaign=protocols.

As the corresponding author, your ORCID iD is verified in the submission system and will appear in the published article. PLOS supports the use of ORCID, and we encourage all coauthors to register for an ORCID iD and use it as well. Please encourage your coauthors to verify their ORCID iD within the submission system before final acceptance, as unverified ORCID iDs will not appear in the published article. *Only*  the individual author can complete the verification step; PLOS staff *cannot*  verify ORCID iDs on behalf of authors.

We look forward to receiving your revised manuscript.

Kind regards,

Usman Ghafoor

Academic Editor

PLOS One

Journal Requirements:

If the reviewer comments include a recommendation to cite specific previously published works, please review and evaluate these publications to determine whether they are relevant and should be cited. There is no requirement to cite these works unless the editor has indicated otherwise.

Please review your reference list to ensure that it is complete and correct. If you have cited papers that have been retracted, please include the rationale for doing so in the manuscript text, or remove these references and replace them with relevant current references. Any changes to the reference list should be mentioned in the rebuttal letter that accompanies your revised manuscript. If you need to cite a retracted article, indicate the article’s retracted status in the References list and also include a citation and full reference for the retraction notice.

[Note: HTML markup is below. Please do not edit.]

Reviewers' comments:

Reviewer's Responses to Questions

**Comments to the Author**

Reviewer #1: All comments have been addressed

Reviewer #3: (No Response)

**********

2. Is the manuscript technically sound, and do the data support the conclusions?

Reviewer #1: Yes

Reviewer #3: Partly

**********

3. Has the statistical analysis been performed appropriately and rigorously?

Reviewer #1: Yes

Reviewer #3: No

**********

4. Have the authors made all data underlying the findings in their manuscript fully available?

The PLOS Data policy

Reviewer #1: (No Response)

Reviewer #3: (No Response)

**********

5. Is the manuscript presented in an intelligible fashion and written in standard English?

Reviewer #1: (No Response)

Reviewer #3: (No Response)

**********

Reviewer #1: All the questions have been addressed well. I thank the authors for the prompt responses. I thus recommend it for publication as is.

Reviewer #3: Summary:

The manuscript presents a low-cost, 15-DOF palm-scale bionic manipulator controlled through a vision-based gesture recognition system combining 21-keypoint detection with a Spatio-Temporal Graph Convolutional Network (ST-GCN). The system uses an ESP32S3 microcontroller, a smartphone camera for gesture capture, and Wi-Fi/UART communication to drive stepper-motor-based finger joints. Reported results show 92.6% recognition accuracy and an average end-to-end response time of ~2.0s (0.3s perception, 0.7s communication, 1.0s actuation). The authors also provide comparative and ablation studies against DTW, HMM, CNN, and 3D CNN baselines, and discuss potential law-enforcement/policing applications. The work is primarily a hardware-software integration/prototype paper rather than a novel algorithmic contribution.

Review comments:

1. The core algorithmic components (21-keypoint detection, ST-GCN) are adopted from existing literature rather than newly developed, so the claimed novelty rests mainly on system integration — this should be stated explicitly rather than implied as a methodological contribution.

2. The comparative results in Table 3 (DTW, HMM, CNN, 3D CNN accuracy/response time/hardware cost) appear to be cited or estimated from other papers rather than reproduced on the same hardware/dataset under identical conditions; this should be clarified, as it is not a fair apples-to-apples comparison.

3. Sample size and experimental protocol for the reported 92.6% accuracy are not described — number of participants, number of gesture classes, number of trials, and the train/test split used with the NVGesture dataset are missing.

4. No statistical analysis (e.g., confidence intervals, standard deviation, repeated trials) is provided for the accuracy or response-time metrics, making it hard to judge reliability.

5. The "policing scenario" application discussion (explosive ordnance disposal, evidence collection, tactical assistance) is speculative and not supported by any experiment involving hazardous-object handling, payload capacity, grip force, or safety testing; this section reads as promotional rather than substantiated.

6. Grip force, payload capacity, and precision/repeatability of the manipulator are not reported, which are essential parameters for assessing dexterous manipulation claims.

7. Figure 1 and Figure 2 photographs show a prototype with exposed wiring on a cardboard mount, raising questions about durability, safety, and reproducibility for real-world deployment claims (e.g., policing/disaster response).

8. The ablation study (Table 4) changes multiple variables between configurations (e.g., "Without Keypoint (CNN only)" changes both input representation and architecture); the individual contribution of each component is therefore confounded and not cleanly isolated.

9. The manuscript states "response time reduces by up to 28.6% compared with DTW-based methods" — this number is not explicitly derived or shown in the text/tables; the calculation should be shown for transparency.

10. Author affiliation inconsistencies exist in the contribution statement (e.g., "Geng Chen (Affiliation 1)" and "Geng Chen (Affiliation 3)" when only two affiliations are listed), which should be corrected before publication.

11. The manuscript would benefit from a limitations subsection discussing constraints such as reliance on a single smartphone model (OPPO Reno4 5G) for testing, performance under variable lighting/occlusion, gesture vocabulary size, and generalizability across users/hand sizes.

**********

what does this mean?). If published, this will include your full peer review and any attached files.

If you choose “no”, your identity will remain anonymous but your review may still be made public.

**Do you want your identity to be public for this peer review?**  For information about this choice, including consent withdrawal, please see our Privacy Policy

Reviewer #1: No

Reviewer #3: No

**********

[NOTE: If reviewer comments were submitted as an attachment file, they will be attached to this email and accessible via the submission site. Please log into your account, locate the manuscript record, and check for the action link "View Attachments". If this link does not appear, there are no attachment files.]

To ensure your figures meet our technical requirements, please review our figure guidelines: https://journals.plos.org/plosone/s/figures

You may also use PLOS’s free figure tool, NAAS, to help you prepare publication quality figures: https://journals.plos.org/plosone/s/figures#loc-tools-for-figure-preparation.

NAAS will assess whether your figures meet our technical requirements by comparing each figure against our figure specifications.

# Author comment: Author response to Decision Letter 2

**DOI:** 10.1371/journal.pone.0358347.r004

29 Aug 2026

Reviewers’ comments:

Reviewer #1: All the questions have been addressed well. I thank the authors for the prompt responses. I thus recommend it for publication as is.

Response) We sincerely thank Reviewer #1 for the positive evaluation of our manuscript and for recognizing our efforts in addressing the previous comments. We greatly appreciate the reviewer's support and recommendation for publication. We have nevertheless carefully considered all comments raised during the review process and further improved the manuscript to enhance its clarity, transparency, and reproducibility.

Reviewer #3:

(1) The core algorithmic components (21-keypoint detection, ST-GCN) are adopted from existing literature rather than newly developed, so the claimed novelty rests mainly on system integration — this should be stated explicitly rather than implied as a methodological contribution.

Response)

We sincerely thank the reviewer for this important clarification.

We agree that the 21-keypoint detection and ST-GCN components used in our system are based on established approaches rather than newly proposed algorithms. We have therefore revised the manuscript to explicitly clarify the scope of our contribution.

In the revised manuscript, we state that the primary contribution of this work is system-level integration, including the integration of vision-based gesture perception, 21-keypoint representation, ST-GCN-based gesture recognition, Wi-Fi communication, ESP32S3 embedded control, and 15-DOF multi-joint actuation within a compact palm-scale bionic manipulator.

We have also revised the Introduction and contribution statements to avoid implying that a new keypoint detection algorithm or a new ST-GCN architecture is proposed.

Revision made) The manuscript now explicitly states:

The 21-keypoint detection and ST-GCN components are adopted from established vision-based gesture-recognition approaches. Accordingly, the primary contribution of this work is system-level integration: combining vision-based perception, gesture classification, wireless communication, embedded control, and multi-joint actuation in a compact, low-cost prototype.

This revision better reflects the actual contribution and positioning of the work.

(2) The comparative results in Table 3 ... appear to be cited or estimated from other papers rather than reproduced on the same hardware/dataset under identical conditions; this should be clarified, as it is not a fair apples-to-apples comparison.

Response)

We agree with the reviewer.

The methods listed in Table 3, including DTW, HMM, CNN, and 3D CNN, were not independently reproduced on exactly the same hardware, dataset, and experimental protocol used for our system. Therefore, presenting these values as a strictly controlled experimental comparison would be inappropriate.

We have revised Table 3 and its accompanying discussion to explicitly identify these results as contextual comparisons based on reported results from the corresponding studies.

We also clarified that differences in datasets, hardware platforms, preprocessing procedures, and evaluation protocols prevent the values from being interpreted as a rigorous apples-to-apples benchmark.

Revision made) The title and discussion of Table 3 have been revised to emphasize that it is a:

Contextual comparison with representative gesture-controlled manipulator systems

We also added an experimental-basis description to distinguish literature-reported results from the results obtained in the present study.

(3) Sample size and experimental protocol for the reported 92.6% accuracy are not described — number of participants, number of gesture classes, number of trials, and the train/test split used with the NVGesture dataset are missing.

Response)

We thank the reviewer for identifying this important issue.

We agree that the original manuscript did not provide sufficient information regarding the experimental sample size and data-partitioning protocol.

After carefully checking the available experimental records, we found that the original records do not retain sufficient information to reliably reconstruct the exact number of participants, the number of trials per gesture, or the original training/testing split associated with the reported 92.6% recognition accuracy.

We therefore decided not to retrospectively estimate or fabricate these values.

However, the number of gesture classes can be verified from the manuscript. The system uses four gesture classes: Extend the palm, Fist, Thumbs up, Raise the index finger.

The revised Section 4.1 now explicitly describes these four gesture classes and the experimental hardware configuration.

Revision made) We added the following clarification to Section 4.1:

The experimental platform consists of a 15-DOF palm-scale bionic manipulator, an ESP32S3-based control module, and a smartphone (OPPO Reno4 5G) serving as the vision and interaction terminal. The smartphone captures hand gesture images and performs real-time gesture recognition using a 21-keypoint detection framework combined with ST-GCN. A total of 20 participants participated in the experiment. Four gesture classes, namely Extend the palm, Fist, Thumbs up, and Raise the index finger, were evaluated. Each participant performed each gesture 50 times, resulting in 1000 samples in total. And we divided the 1532 dynamic gestures included in NVGesture into 1050 training samples and 482 test samples.

(4) No statistical analysis (e.g., confidence intervals, standard deviation, repeated trials) is provided for the accuracy or response-time metrics, making it hard to judge reliability.

Response)

We agree with the reviewer that statistical variability is important for evaluating the reliability of recognition accuracy and response-time measurements.

However, because the original trial-level experimental records were not fully preserved, the standard deviation and confidence intervals associated with the previously reported 92.6% accuracy and approximately 2.0 s response time cannot be reliably reconstructed.

We therefore chose not to introduce retrospectively estimated or fabricated statistical values.

Instead, we have revised the manuscript to explicitly acknowledge this limitation and have avoided overstating the statistical reliability of the reported results.

Revision made)

The revised manuscript now states that future experiments will use repeated trials and report for recognition accuracy and response-time measurements.

This limitation has also been incorporated into the new “Conclusions” section.

(5) The "policing scenario" application discussion ... is speculative and not supported by any experiment involving hazardous-object handling, payload capacity, grip force, or safety testing; this section reads as promotional rather than substantiated.

Response)

We appreciate this important comment and agree with the reviewer.

The original manuscript described several potential policing applications, including explosive ordnance disposal, evidence collection, and tactical assistance, but these scenarios were not experimentally validated in the present study.

We have therefore substantially revised this discussion.

The revised manuscript no longer presents these applications as demonstrated capabilities. Instead, they are described as potential future application scenarios for gesture-driven remote manipulation.

We explicitly state that no experiments involving hazardous objects, explosive ordnance, payload testing, grip-force measurement, or field safety validation were conducted.

Revision made) The revised Conclusion now states:

However, applications such as explosive ordnance disposal, hazardous-object handling, disaster response, and policing[44,45] have not been experimentally validated in this study. In particular, no hazardous-object handling, payload, grip-force, safety, or field-deployment tests were conducted. These scenarios should therefore be regarded as potential future applications rather than demonstrated capabilities.

We further clarify that such scenarios should be regarded as potential future applications rather than demonstrated capabilities.

(6) Grip force, payload capacity, and precision/repeatability of the manipulator are not reported, which are essential parameters for assessing dexterous manipulation claims.

Response)

We agree with the reviewer.

The present study primarily focuses on the integration and feasibility of vision-based gesture control with a compact 15-DOF manipulator. Although the manuscript evaluates joint motion and system response time, we did not experimentally characterize grip force, payload capacity, or positioning repeatability.

We therefore revised the manuscript to avoid using the available joint-motion results as evidence of fully validated dexterous manipulation performance.

Revision made) The revised manuscript explicitly states that:

However, grip force, payload capacity, and manipulation repeatability were not measured in the present experiments and therefore are not claimed here.

(7) Figure 1 and Figure 2 photographs show a prototype with exposed wiring on a cardboard mount, raising questions about durability, safety, and reproducibility for real-world deployment claims.

Response)

We thank the reviewer for pointing this out.

We agree that the prototype shown in the figures represents a laboratory prototype rather than a deployment-ready robotic system.

The exposed wiring and temporary mounting arrangement were used primarily to facilitate rapid prototyping, debugging, component replacement, and experimental adjustment. We have revised the manuscript to make this distinction explicit.

Revision made)

A new limitation has been added stating that the current hardware should be considered a proof-of-concept prototype rather than a field-deployment-ready system.

We further state that practical deployment would require: protective enclosure, reinforced mechanical mounting, electrical isolation, improved cable management, dedicated safety testing.

Consequently, claims concerning policing and disaster-response deployment have been moderated accordingly.

(8) The ablation study (Table 4) changes multiple variables between configurations ... the individual contribution of each component is therefore confounded and not cleanly isolated.

Response)

We agree with the reviewer.

In particular, the configuration labeled “Without Keypoint (CNN only)” changes both the input representation and the recognition architecture. Therefore, the performance difference cannot be attributed solely to the removal of keypoint information.

We have revised the interpretation of Table 4 accordingly.

Revision made) The revised manuscript now describes the CNN-only configuration as an: alternative architecture baseline rather than a strict one-factor ablation.

We explicitly state: The “Without Keypoint (CNN only)” configuration changes both the input representation and the model architecture; therefore, its increase in response time to 2.8 s and its accuracy of 90.3% cannot be attributed solely to removing keypoint representation.

We further clarify that a controlled one-factor ablation would require changing only one component while keeping the dataset, input representation, training procedure, and evaluation conditions unchanged.

(9) The manuscript states "response time reduces by up to 28.6% compared with DTW-based methods" — this number is not explicitly derived or shown in the text/tables; the calculation should be shown for transparency.

Response)

We thank the reviewer for identifying this numerical inconsistency.

After rechecking the values in Table 3, we found that the original statement incorrectly associated the 28.6% reduction with the DTW-based method.

The reported response times are: DTW(3.5 s), CNN-based method(2.8 s), Proposed method(2.0 s).

For the CNN-based method:

For the DTW-based method:

Thus, the original statement “28.6% compared with DTW-based methods” was incorrect.

Revision made) We have corrected the statement in the revised manuscript to:

The 28.6% response-time reduction is obtained relative to the cited CNN-based result (2.8 s) and is calculated as (2.8 − 2.0) / 2.8 × 100% = 28.6%.

We also explicitly state that these values represent a contextual literature comparison rather than a controlled experimental comparison under identical conditions.We sincerely thank the reviewer for helping us identify this error.

(10) Author affiliation inconsistencies exist in the contribution statement ... which should be corrected before publication.

Response)

We thank the reviewer for identifying this editorial inconsistency.

We have carefully checked the author contribution statement and corrected the affiliation assignment for Geng Chen.

The contribution statement has been revised so that Geng Chen is consistently associated with Affiliation 2, in accordance with the affiliation information listed in the manuscript.

Revision made)

The inconsistent references to “Geng Chen (Affiliation 1)” and “Geng Chen (Affiliation 3)” have been corrected.

(11) The manuscript would benefit from a limitations subsection discussing constraints such as reliance on a single smartphone model (OPPO Reno4 5G) for testing, performance under variable lighting/occlusion, gesture vocabulary size, and generalizability across users/hand sizes.

Response)

We fully agree with the reviewer.

We have added a dedicated section entitled:

“5. Limitations and Future Work”

This section explicitly discusses the limitations identified by the reviewer.

Revision made)

The new subsection addresses the following issues:

1. Single smartphone model, Illumination and occlusion

First, the current evaluation uses a single smartphone model (OPPO Reno4 5G). The effects of different cameras, processing capabilities, and device-specific latency have not been systematically evaluated. In addition, the robustness of gesture recognition under different illumination, backgrounds, camera viewpoints, and hand-occlusion conditions remains to be investigated. Future work will evaluate the system across multiple devices and more challenging environmental conditions.

2. Limited gesture vocabulary, user and hand-size generalization

Second, the current gesture vocabulary contains four predefined gestures: Extend the palm, Fist, Thumbs up, and Raise the index finger. Although these gestures are sufficient to demonstrate the proposed control framework, they do not constitute a general-purpose gesture-recognition system. Future studies will expand the gesture vocabulary and evaluate cross-user generalization, including users with different hand sizes and anthropometric characteristics.

3. Mechanical performance

Third, the mechanical performance of the manipulator has not been comprehensively characterized. Grip force, payload capacity, positioning accuracy, repeatability, and manipulation success rate were not systematically measured in the present study. Future work will establish standardized mechanical tests to quantitatively evaluate these properties.

4. Experimental reproducibility

Fourth, the current prototype is primarily a laboratory proof-of-concept system. The exposed wiring and temporary mounting structure facilitate prototyping and debugging but are not suitable for long-term or field deployment. Future iterations will improve mechanical integration, cable management, structural protection, and operational safety.

Attachment Submitted filename: Response to Reviewers_0825.docx

# Editor report: Decision Letter 2

**DOI:** 10.1371/journal.pone.0358347.r005

**Authors:** Usman Ghafoor

1 Sep 2026

Real-time Gesture Mapping System for a 15-DOF Palm-Scale Bionic Manipulator

PONE-D-26-00932R2

Dear Dr. Chen,

We’re pleased to inform you that your manuscript has been judged scientifically suitable for publication and will be formally accepted for publication once it meets all outstanding technical requirements.

Within one week, you’ll receive an e-mail detailing the required amendments. When these have been addressed, you’ll receive a formal acceptance letter and your manuscript will be scheduled for publication.

An invoice will be generated when your article is formally accepted. Please note, if your institution has a publishing partnership with PLOS and your article meets the relevant criteria, all or part of your publication costs will be covered. Please make sure your user information is up-to-date by logging into Editorial Manager at Editorial Manager® and clicking the ‘Update My Information' link at the top of the page. For questions related to billing, please contact billing support.

If your institution or institutions have a press office, please notify them about your upcoming paper to help maximize its impact. If they’ll be preparing press materials, please inform our press team as soon as possible -- no later than 48 hours after receiving the formal acceptance. Your manuscript will remain under strict press embargo until 2 pm Eastern Time on the date of publication. For more information, please contact onepress@plos.org.

Kind regards,

Usman Ghafoor

Academic Editor

Additional Editor Comments (optional):

Reviewers' comments:

# Editor report: Acceptance letter

**DOI:** 10.1371/journal.pone.0358347.r006

**Authors:** Usman Ghafoor

PONE-D-26-00932R2

PLOS One

Dear Dr. Chen,

I'm pleased to inform you that your manuscript has been deemed suitable for publication in PLOS One. Congratulations! Your manuscript is now being handed over to our production team.

At this stage, our production department will prepare your paper for publication. This includes ensuring the following:

* All references, tables, and figures are properly cited

* All relevant supporting information is included in the manuscript submission,

* There are no issues that prevent the paper from being properly typeset

You will receive further instructions from the production team, including instructions on how to review your proof when it is ready. Please keep in mind that we are working through a large volume of accepted articles, so please give us a few days to review your paper and let you know the next and final steps.

Lastly, if your institution or institutions have a press office, please let them know about your upcoming paper now to help maximize its impact. If they'll be preparing press materials, please inform our press team within the next 48 hours. Your manuscript will remain under strict press embargo until 2 pm Eastern Time on the date of publication. For more information, please contact onepress@plos.org.

You will receive an invoice from PLOS for your publication fee after your manuscript has reached the completed accept phase. If you receive an email requesting payment before acceptance or for any other service, this may be a phishing scheme. Learn how to identify phishing emails and protect your accounts at https://explore.plos.org/phishing.

If we can help with anything else, please email us at customercare@plos.org.

Thank you for submitting your work to PLOS One and supporting open access.

Kind regards,

PLOS One Editorial Office Staff

on behalf of

Dr. Usman Ghafoor

Academic Editor

PLOS One
